# Sexually transmitted infections, sexual life and risk behaviours of people living with schizophrenia: systematic review and meta-analysis

**DOI:** 10.1192/bjo.2024.49

**Published:** 2024-05-10

**Authors:** Claudia Aymerich, Borja Pedruzo, Gonzalo Salazar de Pablo, Lander Madaria, Javier Goena, Vanessa Sanchez-Gistau, Paolo Fusar-Poli, Philip McGuire, Miguel Ángel González-Torres, Ana Catalan

**Affiliations:** Psychiatry Department, Basurto University Hospital, Osakidetza, Basque Health Service, Bilbao, Spain; Biobizkaia Health Research Institute, OSI Bilbao-Basurto, Bilbao, Spain; Centro de Investigación en Red de Salud Mental (CIBERSAM), Madrid, Spain; and Neuroscience Department, University of the Basque Country, Leioa, Spain; Psychiatry Department, Basurto University Hospital, Osakidetza, Basque Health Service, Bilbao, Spain; Child and Adolescent Mental Health Services, South London and the Maudsley NHS Foundation Trust, London, UK; Institute of Psychiatry and Mental Health. Department of Child and Adolescent Psychiatry, Hospital General Universitario Gregorio Marañón, School of Medicine, Universidad Complutense, Instituto de Investigación Sanitaria Gregorio Marañón, CIBERSAM, Madrid, Spain; and Department of Child and Adolescent Psychiatry, Institute of Psychiatry, Psychology and Neuroscience, London, UK; Psychiatry Department, Basurto University Hospital, Osakidetza, Basque Health Service, Bilbao, Spain; and Biobizkaia Health Research Institute, OSI Bilbao-Basurto, Bilbao, Spain; Early Intervention in Psychosis Service, Hospital Universitari Institut Pere Mata, IISPV-CERCA, CIBERSAM, ISCIII, Universitat Rovira i Virgili, Reus, Spain; Early Psychosis: Interventions and Clinical-Detection Lab, Department of Psychosis Studies, Institute of Psychiatry, Psychology & Neuroscience, King's College London, London, UK; Department of Brain and Behavioral Sciences, University of Pavia, Pavia, Italy; OASIS Service, South London and Maudsley National Health Service Foundation Trust, London, UK; and National Institute for Health Research, Maudsley Biomedical Research Centre, South London and Maudsley NHS Foundation Trust, London, UK; Department of Psychiatry, University of Oxford, Oxford, UK; and NIHR Oxford Health Biomedical Research Centre, Oxford, UK; Psychiatry Department, Basurto University Hospital, Osakidetza, Basque Health Service, Bilbao, Spain; Biobizkaia Health Research Institute, OSI Bilbao-Basurto, Bilbao, Spain; CIBERSAM, Madrid, Spain; and Neuroscience Department, University of the Basque Country, Leioa, Spain; Psychiatry Department, Basurto University Hospital, Osakidetza, Basque Health Service, Bilbao, Spain; Biobizkaia Health Research Institute, OSI Bilbao-Basurto, Bilbao, Spain; CIBERSAM, Madrid, Spain; Neuroscience Department, University of the Basque Country, Leioa, Spain; Early Psychosis: Interventions and Clinical-Detection Lab, Department of Psychosis Studies, Institute of Psychiatry, Psychology & Neuroscience, King's College London, London, UK; and Department of Psychiatry, University of Oxford, Oxford, UK

**Keywords:** Psychotic disorders/schizophrenia, STI, sexual life, contraception, HIV

## Abstract

**Background:**

Sexually transmitted infections (STIs), along with sexual health and behaviour, have received little attention in schizophrenia patients.

**Aims:**

To systematically review and meta-analytically characterise the prevalence of STIs and sexual risk behaviours among schizophrenia patients.

**Method:**

Web of Science, PubMed, BIOSIS, KCI-Korean Journal Database, MEDLINE, Russian Science Citation Index, SciELO and Cochrane Central Register were systematically searched from inception to 6 July 2023. Studies reporting on the prevalence or odds ratio of any STI or any outcome related to sexual risk behaviours among schizophrenia samples were included. PRISMA/MOOSE-compliant (CRD42023443602) random-effects meta-analyses were used for the selected outcomes. Q-statistics, *I*^2^ index, sensitivity analyses and meta-regressions were used. Study quality and publication bias were assessed.

**Results:**

Forty-eight studies (*N* = 2 459 456) reporting on STI prevalence (including 15 allowing for calculation of an odds ratio) and 33 studies (*N* = 4255) reporting on sexual risk behaviours were included. Schizophrenia samples showed a high prevalence of STIs and higher risks of HIV (odds ratio = 2.11; 95% CI 1.23–3.63), hepatitis C virus (HCV, odds ratio = 4.54; 95% CI 2.15–961) and hepatitis B virus (HBV; odds ratio = 2.42; 95% CI 1.95–3.01) infections than healthy controls. HIV prevalence was higher in Africa compared with other continents and in in-patient (rather than out-patient) settings. Finally, 37.7% (95% CI 31.5–44.4%) of patients were sexually active; 35.0% (95% CI 6.6–59.3%) reported consistent condom use, and 55.3% (95% CI 25.0–82.4%) maintained unprotected sexual relationships.

**Conclusions:**

Schizophrenia patients have high prevalence of STIs, with several-fold increased risks of HIV, HBV and HCV infection compared with the general population. Sexual health must be considered as an integral component of care.

The World Health Organization defines sexual health as ‘a state of physical, emotional, mental, and social well-being in sexuality’.^1^ Sexuality is a natural aspect of human behaviour and a significant factor in quality of life and maintaining healthy relationships.^2^ However, for individuals living with schizophrenia, sexual health has received little attention or recognition as a fundamental aspect of their subjective quality of life and associated care.^3^ Data suggest that people with schizophrenia have both quantitative and qualitative differences in their sexual lives compared with the general population,^4^ identifying this area of health as one with unmet needs,^5^ although sexual interest and activity do not disappear after diagnosis.^6,7^

Indeed, individuals with schizophrenia are at a higher risk of engaging in risky sexual behaviors,^7^ with potentially harmful physical and mental health consequences such as unwanted pregnancies,^8^ exposure to interpersonal violence in relationships^9^ and increased prevalence of sexually transmitted infections (STIs).^10^ The relationship between STIs and schizophrenia is complex and multifactorial, with an increase of risk of STIs due to psychiatric symptoms (e.g. disorganised behaviour leading to hypersexuality^11^ or negative symptoms leading to a lack of skills to assertively negotiate safer relationships^12^). Severe stigmatisation, particularly in romantic relationships,^13^ and high rates of comorbidity with other mental disorders and substance use,^14,15^ among many other factors, also contribute to this problem. On the other hand, early exposure to certain microorganisms such as hepatitis C virus (HCV)^16^ or chlamydia^17^ is associated with a higher risk of developing schizophrenia.^18,19^ Comorbidity between schizophrenia and viral diseases leads to a poorer prognosis for both conditions.^20^

Despite all the above findings, the sexual lives and risky behaviours of individuals living with severe mental health disorders in general, and schizophrenia in particular, continue to be neglected both in clinical practice and research. There is a significant knowledge gap in the available literature, in contrast to other important aspects of quality of life.^4^

Considering these complexities, this systematic review and meta-analysis aimed to fill this gap and examine the prevalence of STIs in this population, their increased risk compared with the general population, and the demographic, clinical and methodological factors influencing this risk. Second, we aimed to characterise the sexual risk behaviours associated with schizophrenia.

## Method

This study protocol was registered on PROSPERO (registration number: CRD42023443602). The study was conducted in accordance with the PRISMA (Preferred Reporting Items for Systematic Reviews and Meta-Analyses)^21^ (Supplementary Table 1 available at https://doi.org/10.1192/bjo.2024.49) and MOOSE (Meta-Analyses of Observational Studies in Epidemiology^22^ (Supplementary Table 2) checklists, following the EQUATOR reporting guidelines.^23^

### Search strategy and selection criteria

A systematic literature search was carried out dually and independently by two investigators (C.A. and B.P.). The search encompassed the Web of Science database (Clarivate Analytics), including the Web of Science Core Collection, PubMed, the BIOSIS Citation Index, the KCI-Korean Journal Database, MEDLINE, the Russian Science Citation Index, and the SciELO Citation Index, as well as the Cochrane Central Register of Reviews and Ovid/PsycINFO databases, from inception until 6 July 2023. Two separated searches were conducted: one to identify articles containing information on the prevalence and relative risk of sexually transmitted diseases among people with a diagnosis of schizophrenia spectrum disorder, and the other to identify articles reporting on outcomes related to sexual behaviour among the same population. The complete search terms are available in Supplementary Table 3.

Articles identified underwent an initial screening of their abstracts by the two reviewers. Subsequently, after exclusion of those that did not meet the inclusion criteria, the full texts of the remaining articles were dually assessed for eligibility and inclusion. Inclusion criteria for the systematic review and meta-analysis were: (a) individual studies with original data; (b) reporting on patients meeting criteria for any schizophrenia spectrum disorder (including schizophrenia, schizophreniphorm disorder, schizoaffective disorder, delusional disorder, and brief psychotic disorder, according to DSM-5-TR^24^ or ICD-11^25^ criteria); (c) reporting either quantitative data on the prevalence of an STI (including HIV, human papillomavirus, hepatitis B virus (HBV), HCV, *Treponema pallidum*, *Neisseria gonorrhoeae*, *Mycoplasma genitalium* and *Chlamydia trachomatis*) using a serological, microbiological or clinical diagnosis provided by a healthcare specialist, or either any outcome related to sexual behaviour (a complete list of the sought-out, standardised outcomes is available in Supplementary Table 4); (d) non-overlapping samples (overlap was ascertained by examining the inclusion dates, the demographics of the population and the country where the study was conducted; the study with the largest sample was selected); and (e) written in the English language. Exclusion criteria were (a) reviews, clinical cases, study protocols or qualitative studies, conferential proceedings, letters and commentaries; (b) reporting on patients with an affective psychotic disorder according to DSM/ICD criteria;^24,25^ (c) reporting on a subsample of schizophrenia patients specifically selected for their characteristics or risk of an STI; and (d) written in languages other than English.

### Data extraction

Three reviewers (B.P., L.M. and J.G.) independently conducted data extraction from all the studies included, starting on 20 July 2023. Subsequently, the three databases were cross-checked, and any inconsistencies were resolved through consensus under the supervision of a senior researcher (A.C.).

For the included articles, a summary of the selected variables included: first author and year of publication, country and city, sample size, age in years (mean ± s.d.), sex (percentage female), STI diagnostic method, relationship status (percentage in stable relationship), substance use disorder according to any DSM or ICD criteria (excluding nicotine) (%), quality assessment (see below) and key findings. When stratified data were available, data were extracted separately for male and female populations.

### Risk of bias (quality) assessment

Risk of bias was independently assessed by B.P. and C.A. using a modified version of the Newcastle–Ottawa Scale (NOS) for assessing the quality of non-randomised studies. This choice was made taking into account the heterogeneity expected in the included studies^26^ (Supplementary Table 5). Any discrepancy between the two assessments was resolved through consensus.

### Strategy for data synthesis

First, we provided a systematic synthesis of the findings from the included studies structured around two main topics: the prevalence and relative risk of the examined STIs, and the included sex behaviour outcomes (Table 1 and Supplementary Table 6, respectively).

**Table 1:** Characteristics of the studies included in the sexually transmitted infections systematic review

Study	Country	STI	*N*, schizophrenia patients (STI)	*N*, healthy controls (STI)	Age in years, mean (s.d.)	Percentage women	Setting	Percentage with SUD	Percentage in stable relationship	NOS
Opondo et al, 2017	Botswana	HIV	545 (152)	–	30.3 (3.8)	22%	In-patient	n.a.	5%	7
Said et al, 2001	Jordan	HBV	192 (14)	192 (5)	39.9 (n.a.)	45%	In-patient	n.a.	n.a.	6
Doufik et al, 2022	Morocco	HIV	444 (0)	–	33.5 (9.2)	10%	Other	n.a.	24%	5
		HBV	444 (7)	–						
		HCV	444 (4)	–						
		*T. pallidum*	444 (16)	–						
Mona et al, 2022	South Africa	HIV	370 (45)	–	n.a.	30%	In-patient	n.a.	6%	7
Mwelase et al, 2023	South Africa	HIV	294 (62)	–	n.a.	31%	In-patient	53%	58%	8
Lundberg et al, 2013	Uganda	HIV	224 (26)	15 108 (1330)	n.a.	51%	Other	n.a.	14%	8
Maling et al, 2011	Uganda	HIV	87 (13)	–	n.a.	n.a.	In-patient	n.a.	n.a.	8
Mbewe et al, 2006	Zambia	HIV	160 (5)	–	37.5 (21.4)	28%	In-patient	56%	n.a.	6
Wang et al, 2016	China	HBV	415 (28)	3038 (101)	18.5 (1.6)	48%	Other	n.a.	n.a.	6
Zhan et al, 2018	China	*T. pallidum*	1586 (53)	–	n.a.	n.a.	Other	n.a.	n.a.	8
Zhu et al, 2015	China	HBV	1649 (181)	–	34.0 (n.a.)	54%	Other	n.a.	n.a.	7
Chaudury et al, 1994	India	HBV	100 (11)	100 (2)	54.6 (8.4)	0%	In-patient	n.a.	n.a.	4
Imani et al, 2022	Iran	HBV	92 (1)	–	n.a.	n.a.	Other	n.a.	n.a.	7
Nakamura et al, 2004	Japan	HCV	455 (28)	197 827 (2374)	n.a.	n.a.	In-patient	n.a.	n.a.	8
Chang et al, 2021	Taiwan	HBV	15 914 (465)	–	40.1 (9.7)	n.a.	Other	n.a.	n.a.	9
		HCV	15 914 (181)	–						
Chiu et al, 2017	Taiwan	HCV	6097 (127)	6097 (85)	43.3 (13.7)	48%	Other	2%	n.a.	7
Hung et al, 2012	Taiwan	HBV	511 (53)	–	42.5 (10.7)	42%	In-patient	n.a.	n.a.	8
		HCV	577 (11)	–						
Hariri et al, 2011	Turkey	HIV	88 (0)	–	34.9 (8.8)	64%	Out-patient	n.a.	n.a.	8
		HBV	88 (0)	–						
		HCV	88 (0)	–						
De Hert et al, 2009	Belgium	HIV	595 (3)	–	36.7 (11.2)	35%	Other	n.a.	13%	7
		HCV	595 (4)	–						
Fellerhoff et al, 2011	Germany	*C. trachomatis*	72 (2)	−	n.a.	n.a.	Other	n.a.	n.a.	7
Krause et al, 2010	Germany	*C. trachomati*s	31 (8)	−	n.a.	n.a.	Other	n.a.	n.a.	7
Grassi et al, 1999	Italy	HIV	33 (1)	−	35.3 (8.1)	35%	Other	43%	15%	6
Cuadrado et al, 2020	Spain	HCV	425 (8)	−	36.5 (n.a.)	47%	Other	n.a.	n.a.	7
González-Torres et al, 2015	Spain	HIV	235 (5)	−	n.a.	n.a.	In-patient	n.a.	n.a.	7
Bauer-Staeb, 2017	Sweden	HIV	21 232 (44)	6 815 931 (5909)	46.0 (8.1)	50%	Other	4%	n.a.	8
		HBV	21 232 (112)	6 815 931 (112)						
		HCV	21 232 (1194)	6 815 931 (41 600)						
Jallow et al, 2016	Sweden	HIV	10 347 (65)	−	n.a.	46%	Out-patient	n.a.	n.a.	8
Karabulut et al, 2016	Turkey	HIV	489 (0)	−	42.5 (11.3)	16%	Other	n.a.	n.a.	7
		HBV	489 (32)	−						
		HCV	489 (1)	−						
Heslin et al, 2022	United Kingdom	HIV	8562 (174)	−	n.a.	n.a.	Out-patient	n.a.	n.a.	9
Closson et al, 2019	Canada	HIV	6454 (835)	507 670 (12 499)	n.a.	41%	Other	49%	n.a.	8
Sockalingam et al, 2010	Canada	HCV	110 (3)	−	44.7 (10.8)	32%	Other	7%	n.a.	7
Rodgers-Johnson et al, 1996	Jamaica	HIV	201 (5)	−	n.a.	38%	In-patient	n.a.	17%	7
Alvarado-Esquivel et al, 2005	Mexico	HBV	33 (4)	–	n.a.	n.a.	In-patient	n.a.	n.a.	7
Baillargeon et al, 2008	USA	HIV	4736 (173)	–	n.a.	n.a.	Other	n.a.	n.a.	5
Blank et al, 2002	USA	HIV	8208 (98)	374 253 (2062)	40.3 (17.6)	47%	Other	n.a.	n.a.	6
Carney et al, 2006	USA	HCV	1074 (7)	726 262 (492)	40.2 (11.9)	53%	Other	9%	n.a.	7
Dinwiddie et al, 2003	USA	HCV	153 (14)	–	n.a.	n.a.	In-patient	n.a.	n.a.	7
Doyle et al, 1997	USA	HIV	138 (0)	–	32.0 (13.7)	n.a.	In-patient	27%	n.a.	5
Freudenreich et al, 2007	USA	HCV	98 (8)	–	44.7 (n.a.)	25%	Out-patient	n.a.	n.a.	6
Fuller et al, 2011	USA	HCV	6521 (1076)	6521 (124)	57.2 (n.a.)	6%	Other	65%	n.a.	8
Hart et al, 1999	USA	HIV	38 (2)	16 (0)	n.a.	n.a.	Other	n.a.	n.a.	4
Himelhoch et al, 2007	USA	HIV	89 189 (858)	67 965 (346)	55.5 (11.9)	5%	Other	25%	25%	7
		HCV	89 189 (6287)	67 965 (1708)						
Huckans et al, 2006	USA	HCV	2207 (219)	73 687 (3888)	n.a.	n.a.	Other	n.a.	n.a.	6
Prince et al, 2012	USA	HIV	221 017 (1413)	4 089 407 (24 607)	n.a.	n.a.	Other	n.a.	n.a.	8
Rosengerg et al, 2005	USA	HIV	495 (18)	–	n.a.	32%	Other	29%	n.a.	7
		HCV	495 (96)	–						
Walkup et al, 2010	USA	HIV	2 047 199 (37 054)	–	n.a.	n.a.	Other	n.a.	n.a.	6
Brown et al, 2021	Australia	HIV	69 (1)	–	19.6 (10.8)	42%	Other	n.a.	10%	9
		*C. trachomatis*	69 (5)	–						
Williams et al, 2020	Australia	HIV	97 (0)	–	46.0 (n.a.)	40%	Out-patient	n.a.	n.a.	6
		HBV	97 (0)	–						
		HCV	97 (7)	–						
Santos da Silva et al, 2019	Brazil	HIV	66 (0)	–	n.a.	n.a.	Out-patient	n.a.	n.a.	8
		HBV	66 (1)	–						
		HCV	66 (0)	–						
		*T. pallidum*	66 (0)	–						

STI, sexually transmitted disease; SUD, substance use disorder; NOS, Newcastle–Ottawa Scale; HBV, hepatitis B virus; HCV, hepatitis C virus.

Second, where data allowed, we performed meta-analyses using as primary effect size the prevalence (percentage and standard error, when available) of the STIs. Each STI was separately analysed. Then, for those articles where the prevalence of STIs in a comparison group of healthy controls (defined as people without any mental health disorder) was also available, the odds ratio with a 95% confidence interval was calculated using the number of individuals with any particular STI and samples sizes for each sample, without adjusting by any variable, and then separately meta-analysed for each STI. An odds ratio greater than 1 indicated that the schizophrenia group had a higher risk of presenting with any particular STI than the healthy control group. Separate proportion meta-analyses were also conducted to study the pooled prevalence of each sexual behaviour or risk behaviour when three or more samples were available.

The heterogeneity between studies was measured using the Q-statistic, and percentages of overall variability in the estimates of ORs were determined using the *I*^2^ index, classifying the heterogeneity into low (*I*^2^ = 25%), medium (*I*^2^ = 50%) and high (*I*^2^ = 75%).^27^

Meta-regressions were performed to study the effects of (a) age, (b) publication year, (c) percentage of females, (d) percentage of patients with substance use disorder, (e) percentage of patients in a stable relationship, and (f) risk of bias (NOS score) on outcomes where seven or more articles provided the data. Sensitivity analyses were performed to determine differences depending on (a) sample continent, (b) sample type (first-episode psychosis, defined as patients presenting with psychosis for fewer than 5 years from the initial onset,^24^ versus chronic schizophrenia), and (c) setting (in-patient versus out-patient) with respect to the study outcomes when more than ten articles were available. A random-effects model was used, owing to the expected high heterogeneity. Publication bias was assessed by visual inspection of the funnel plots; when more than ten articles were available, Egger's test was also performed.

All analyses were conducted within R 4.2.2^28^ using the metafor package.^29^ The significance level was set at *P* < 0.05, two-sided.

## Results

### Sexually transmitted diseases

The literature search of electronic databases yielded 1734 citations, which were screened for eligibility; 95 articles underwent full-text assessment, and 47 were excluded. The final sample for the systematic review and STI meta-analyses included 48 studies (Supplementary Fig. 1(a)).

Twenty-eight studies (58.3%) included data on HIV,^30,57^ 20 (41.7%) on HCV,^36,40,45,48,50,51,54,55,57,67^ 14 (29.2%) on HBV,^36,48,50,51,54,57,60,64,68,72^ three (8.3%) on *C. trachomatis*^17,49,73^ and three (6.3%) on *T. pallidum*.^36,48,74^ No studies fulfilling our inclusion criteria were found regarding other STIs included in our search. The full sample included 2 459 456 patients with schizophrenia. The mean age of the sample was 50.3 years, ranging from 16 to 73 years (s.d. = 11.9); 21.1% were female, 24.8% were in a stable relationship, and 23.7% presented with a comorbid substance use disorder other than nicotine-related. Among the studies reporting the prevalence of a comorbid substance use disorder, two reported on alcohol and cannabinoids,^30,32^ four reported on the use of injectable drugs,^41,51,53,58^ four reported on both of these categories,^40,45,49,63^ and six did not specify the substance or substances used.^39,43,54,59,62,66^ Studies included samples from 24 countries in six continents: 17 (35.4%) from North America, ten (20.8%) from Europe, ten (20.8%) from Asia, eight (16.7%) from Africa, two (4.2%) from Oceania and one (2.1%) from South America. The mean NOS score for the included studies was 6.9 ± 1.2 (Table 2A and Supplementary Table 6).

**Table 2A:** Prevalence of sexually transmitted infections

STI	Number of studies	Sample size	Prevalence	95% CI	Heterogeneity	
					*I*^2^ (%)	*P*
HIV	28	2 421 702	0.0167	0.0082–0.0337	99.6	<0.01
HCV	20	146 326	0.0282	0.0151–0.0520	99.0	<0.01
HBV	14	41 322	0.0326	0.0157–0.0664	98.4	<0.01
*C. trachomatis*	3	172	0.0850	0.0069–0.5540	82.4	<0.01
*T. pallidum*	3	2096	0.0329	0.0197–0.0545	0.00	0.96

STI, sexually transmitted infection; HCV, hepatitis C virus; HBV, hepatitis B virus.

Fifteen of the included studies provided data for a healthy control comparison group, thereby enabling the calculation of an odds ratio. Of these studies, seven included data on HIV,^33,38,39,41,44,45,54^ seven on HCV^45,54,61,63,66,67^ and four on HBV^54,69,72,75^ (Table 2B).

**Table 2B:** Odds ratio for the risk of each sexually transmitted infection among schizophrenia samples compared with healthy control samples

STI	Number of studies	Schizophrenia patient sample	Healthy control sample	Odds ratio	95% CI	*P*-value	Heterogeneity	
							*I*^2^ (%)	*P*
HIV	7	346 362	11 870 350	2.11	1.23–3.63	0.01	99.5	0.00
HCV	20	126 775	7 894 290	4.54	2.15–9.61	0.00	99.5	0.00
HBV	4	21 939	6 819 261	2.42	1.95–3.01	0.00	0.00	0.59

STI, sexually transmitted infection; HCV, hepatitis C virus; HBV, hepatitis B virus.

#### HIV

**Fig. 1:**
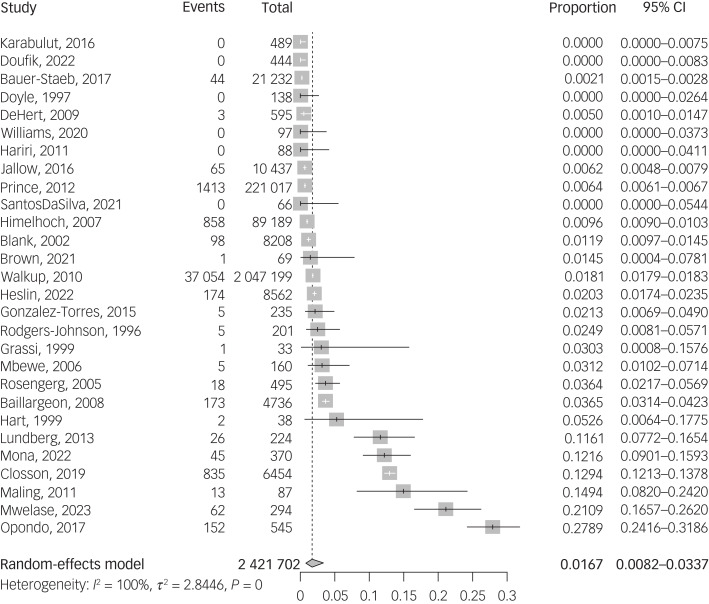
Forest plot of HIV prevalence.

The prevalence of HIV among people with schizophrenia was reported in 28 studies, comprising a total sample of 2 421 702 patients. All HIV diagnosis were serological. The pooled prevalence of HIV was 1.67% (95% CI 0.82–3.37%) (Fig. 1). Meta-regressions found a statistically significant higher prevalence of HIV among samples with higher prevalence of substance use disorder (β = 8.079; 95% CI 0.003–4.020) but no statistically significant effect of age, sex, relationship status, risk of bias or publication year (Supplementary Table 7). Prevalence of HIV was significantly higher in samples from Africa (7.32%; 95% CI 1.51–28.94%) and in in-patient settings (5.94%; 95% CI 1.78–18.04%) when compared with other continents or with out-patient settings (Supplementary Table 9). No publication bias was identified by visual inspection of the funnel plot (Supplementary Fig. 3(a)) or by Egger's test (*P* = 0.48).

**Fig. 2:**
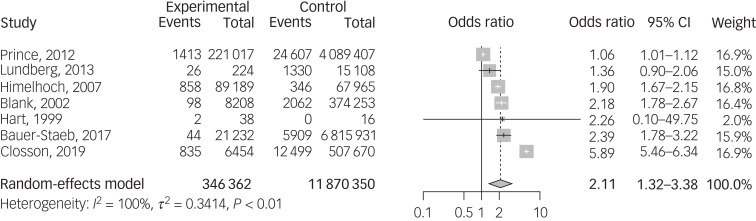
Forest plot of HIV infection odds ratios. An odds ratio greater than 1 implies that the schizophrenia population has greater risk of the infection.

Seven of these studies also included the prevalence of HIV in a healthy control comparison sample (total sample: 346 362 patients with schizophrenia and 11 870 350 healthy controls), allowing for an odds ratio calculation. The odds ratio for HIV infection was 2.11 (95% CI 1.23–3.63, *P* < 0.01), implying a statistically significant higher risk of HIV infection in the schizophrenia sample (Fig. 2). Meta-regressions revealed no statistically significant effect of risk of bias or publication year. The funnel plot did not suggest the presence of publication bias (Supplementary Fig. 3(b)).

#### Hepatitis C virus

The prevalence of HCV among the schizophrenia sample was reported in 20 studies (total sample: 146 326 patients). All diagnosis were serological. The pooled prevalence of HCV was 2.82% (95% CI 1.51–5.20%) (Supplementary Fig. 2(a)). Meta-regressions found a statistically significant higher prevalence of HCV prevalence in older samples (β = 0.143; 95% CI 0.090–0.196) and samples with higher prevalence of substance use disorder (β = 4.201; 95% CI 0.692–7.710) and in older articles (publication year β = −0.097; 95% CI −0.187 to −0.007) (Supplementary Table 8). No effect of setting was detected in the sensitivity analyses (Supplementary Table 10), and no publication bias was identified (Supplementary Fig. 3(c)).

Seven of these studies also included the prevalence of HCV for a healthy control comparison group (total sample: 126 775 patients with schizophrenia and 7 894 290 healthy controls), allowing for an odds ratio calculation. The odds ratio for HCV infection was 4.54 (95% CI 2.15–9.61, *P* < 0.01), implying a statistically significant higher risk of HCV infection in the schizophrenia sample (Supplementary Fig. 2(b)). Meta-regressions revealed no statistically significant effect of risk of bias or publication year, and the funnel plot did not suggest the presence of publication bias (Supplementary Fig. 3(d)).

#### Hepatitis B virus

The prevalence of HBV among people with schizophrenia was reported in 14 studies, comprising a total sample of 41 322 patients. All diagnosis were serological. The pooled prevalence of HBV was 3.26% (95% CI 1.57–6.64%) (forest plot available in Supplementary Fig. 2(c)). Meta-regressions found a statistically significant higher prevalence of HBV prevalence in older articles (publication year β = −0.082; 95% CI −0.157 to −0.007) (Supplementary Table 8), and sensitivity analyses found a greater prevalence of HBV among in-patient samples (9.81%; 95% CI 6.99–13.60%) compared with out-patient or mixed samples (Supplementary Table 10). No publication bias was identified (Supplementary Fig. 3(e)).

Four of these studies also included the prevalence of HBV for a healthy control comparison sample (total sample: 21 939 patients with schizophrenia and 6 819 261 healthy controls), allowing for an odds ratio calculation. The odds ratio for HBV infection was 2.42 (95% CI 1.95–3.01, *P* < 0.01), implying a statistically significant higher risk of HBV infection in the schizophrenia sample (Supplementary Fig. 2(d)). The funnel plot did not suggest the presence of publication bias (Supplementary Fig. 3(f)). Not enough data were available to perform any meta-regression or sensitivity analysis.

#### C. trachomatis

The prevalence of *C. trachomatis* in the schizophrenia sample was reported in three studies (total sample: 172 patients). One article provided clinical diagnosis by the patients’ general practitioners,^49^ another reported serological diagnosis^73^ and the third used molecular diagnosis through DNA polymerase chain reaction.^17^ The pooled prevalence of chlamydia was 8.50% (95% CI 0.69–55.40%) (Supplementary Fig. 2(e)). Not enough data were available to perform any meta-regression or sensitivity analysis, or to calculate an odds ratio for *C. trachomatis* comparing a schizophrenia sample with a healthy control comparison sample.

#### T. pallidum

The prevalence of *T. pallidum* in the schizophrenia sample was reported in three studies (total sample: 2096 patients). All diagnoses were serological. The pooled prevalence of *T. pallidum* was 3.29% (95% CI 1.97–5.45%) (Supplementary Fig. 2(f)). Not enough data were available to perform any meta-regression or sensitivity analysis, or to calculate an OR for *T. pallidum* comparing a schizophrenia sample with a healthy control comparison sample.

### Sexual behaviour

The literature search of electronic databases yielded 789 citations, which were screened for eligibility; full texts of 344 articles were assessed, and 311 articles were excluded. The final sample for the systematic review and meta-analyses included 33 studies (Supplementary Fig. 1(b)).

The full sample comprised 4255 patients with schizophrenia. The mean age of the sample was 38.0 years, ranging from 16 to 65 years (s.d. = 8.02); 51.2% were female, 33.72% declared themselves to be in a stable relationship, and the mean duration of illness was 11.9 years (s.d. = 7.4). Studies included samples from 14 countries in five continents. The mean age at first sexual relationship was 18.15 years. The mean NOS score of the included studies was 6.7 ± 1.2 (Table 2).

A detailed description of the meta-analytical results can be found in Table 2; 37.77% (95% CI 18.93–61.22%) considered themselves to be in a stable relationship.^49,50,53,76,91^ 59.66% (95% CI 43.57–73.91%) reported being interested in sexual relationships with others^4,76,77^ and 53.71% (42.85–64.22%) were satisfied with their sex life.^77,85,92^ Whereas 74.10% (95% CI 53.20–87.89%) had had sexual relationships with another person at least once in their lifetime,^37,49,53,77,79,81,93^ only 37.72% (95% CI 31.52–44.35%) were sexually active (defined in most cases as sexual intercourse at least once over the previous 12 months).^50,53,77,79,84,85,92,94,98^ Among those who were sexually active, 35.37% (95% CI 15.56–61.92%) reported having multiple partners,^50,53,83,84,87,96^ 30.95% (95% CI 11.88–59.84%) had paid for sexual relationships,^50,53,92^ and 13.38% (95% CI 5.02–31.09%) reported having had relationships in exchange for goods or money.^50,83,87^ Only 34.98% (95% CI 16.58–59.29%) reported consistent use of a condom in their relationships,^37,49,53,80,83,96^ whereas 55.28% (95% CI 24.59–82.41%) reported having unprotected sexual relationships,^49,50,80,87,98,99^ and 28.72% (95% CI 8.38–63.99%) of patients had experienced an unplanned pregnancy on the part of themselves or their partners^49,50,92,100,101^ (Table 3A and Supplementary Fig. 2(g,i)). Meta-regressions and sensitivity analyses revealed no statistically significant differences regarding age, sex, risk of bias, publication year, continent or setting for any of the studied outcomes (Supplementary Tables 8 and 10, respectively). The funnel plots did not suggest the presence of publication bias for any of the outcomes (Supplementary Fig. 3).

**Table 3A:** Prevalence of each of the studied sexual and risk behaviours

	Number of studies	Sample size	Prevalence	95% CI	Heterogeneity	
					*I*^2^ (%)	*P*
Stable relationship (%)	20	2127	0.3777	0.1893–0.6122	93.4	<0.01
Lifetime sexual relationship (%)	7	881	0.7410	0.5320–0.8780	91.9	<0.01
Satisfaction with sex life (%)	3	391	0.5371	0.4285–0.6422	49.7	<0.01
Interest in sexual relationship (%)	3	576	0.5966	0.4357–0.7391	70.8	0.03
Sexually active (%)	16	2292	0.3772	0.3152–0.4435	85.9	<0.01
Among sexually active people with schizophrenia						
Prostitution use (%)	3	223	0.3095	0.1188–0.5984	80.8	0.01
Prostitution work (%)	3	612	0.1338	0.0502–0.3109	77.3	0.01
Consistent use of condom (%)	6	577	0.3498	0.1658–0.5929	93.5	<0.01
Hormonal contraception (%)	3	154	0.1297	0.0017–0.9300	94.5	<0.01
Unprotected sexual relationship (%)	6	937	0.5528	0.2459–0.8241	97.9	<0.01
Unplanned pregnancy (%)	5	286	0.2872	0.0838–0.6399	92.0	<0.01
Multiple partners (%)	6	861	0.3537	0.1556–0.6192	97.0	<0.01

When compared with healthy controls, patients with schizophrenia were significantly less likely to be in a stable relationship (*k* = 6, odds ratio = 0.18, 95% CI 0.07–0.45, *P* < 0.01)^49,50,78,79,84,89^ or to be sexually active (*k* = 4, odds ratio 0.19, 95% CI 0.13–0.29, *P* < 0.01)^50,79,84,92^ (Tables 2B and 3B, and Supplementary Fig. 2(h, j)).

**Table 3B:** Odds ratio for the risk of being in a stable relationship and being sexually active among schizophrenia samples compared with healthy controls

	Number of studies	Schizophrenia patient sample	Healthy control sample	Odds ratio	95% CI	*P*-value	Heterogeneity	
							*I*^2^ (%)	*P*
Stable relationship (%)	6	489	518	0.18	0.07–0.45	0.00*	86.5	0.00
Sexually active (%)	4	285	317	0.19	0.13–0.29	0.00*	27.2	0.00

## Discussion

To the best of our knowledge, this is the first systematic review and meta-analysis to comprehensively assess the prevalence and odds ratios of STIs among people living with schizophrenia, along with their sexual risk behaviours.

Several important findings have been made. First, a high prevalence of STIs was noted. The pooled HIV prevalence was 1.67% (with an odds ratio of 2.11 compared with the general population), whereas for HCV and HBV, positivity prevalence reached 2.82 and 3.26%, with ORs of 4.54 and 2.42, respectively. A high prevalence was also been found for less-studied STIs such as *T. pallidum* (3.3%) and *C. trachomatis* (8.5%). It is important to highlight that the included studies were cross-sectional, so it can be anticipated that the proportion of individuals with schizophrenia who develop an STI over the course of their lifetime will be substantially higher than reported here. This is in line with previous findings in literature, from systematic reviews^102^ and large cohort studies.^10,103^ Positive symptoms are associated with disorganised behaviour, substance use (including injection drug use, another major source of contagion for the studied viruses) and hypersexuality in some cases.^87,104^ In our meta-analysis, HIV prevalence was substantially higher in samples with higher substance use disorder comorbidity and in samples from Africa, at 7.32%. A previous meta-analysis examining the prevalence of HIV seropositivity among patients with first-episode psychosis patients in the African continent found an even greater pooled prevalence of 26%, which they hypothetically linked to longer duration of untreated schizophrenia, low access to health services and high prevalence of infection in the continent.^105^ On the other hand, and more encouragingly, the prevalence of HBV and HCV appears to be lower according to more recently published articles (and in the case of HCV, for samples with younger mean age). Global trends for hepatitis B and C have shown a positive evolution over the last decades,^106^ especially with the appearance of direct-acting antiviral treatments for HCV.^107^ This has been especially notorious in some correctional institutions,^108^ where patients with severe mental health disorders are overrepresented.^109^

On the other hand, another important finding of our study was that individuals with schizophrenia were significantly less likely to be in a stable relationship (odds ratio = 0.18) or engage in sexual activity with other people (odds ratio = 0.19) compared with healthy controls. This is consistent with previous findings in the literature, with studies reporting both lower rates of marriage and higher rates of divorce among people with schizophrenia.^110^ Furthermore, the overall pooled prevalence of patients in our study who declared themselves to be sexually active was under 40%. This could be attributed to several factors. Positive symptoms such as sex-related delusions and hallucinations can have a negative impact on relationships and sexual life,^111^ whereas negative symptoms are associated with sexual dysfunction and deficits in sexual interest and activity.^76^ In our meta-analysis, 59.6% of patients (pooled prevalence) reported being interested in maintaining sexual intercourse with other people. Bianco et al reported a bimodal distribution of sexual interest among adults with schizophrenia, with most patients reporting either no problem with sexual interest or severe impairment in that area.^76^ Even when sexual interest is present, sexual dysfunction is a frequent side-effect associated with the use of antipsychotic medications, occurring both directly through elevated prolactin due to blockade of dopamine D2 receptors^112^ and indirectly through other adverse effects such as metabolic syndrome and obesity.^113^ Other sources of sexual dysfunction may include concomitant use of antidepressants and anxiolytics, comorbidity with other mental health and substance use disorders^114^ and, in more severely affected populations, the closed management model of most psychiatric in-patient units, which leads to a lack of privacy and limits the chance of having sexual activity.^5^ It is important to address this, as a satisfactory romantic and sexual life has proven to be beneficial for the recovery of people with schizophrenia, increasing self-confidence, treatment compliance and even overall survival.^5,115^

Among those who were sexually active, a great prevalence of risk behaviours was found. Only 34.9 and 12.9% of patients with schizophrenia reported consistently using condoms or hormonal contraception in their sexual relationships, whereas 55.3% of the pooled sample regularly had unprotected intercourse. Moreover, 35.4% of patients reported having multiple concurrent sexual partners, and 28.7% had experienced an unwanted pregnancy either themselves or in their partners. This pattern of concerning sexual behaviours among people living with schizophrenia has been described in previous studies, with a prevalence of risky practices of up to 83%.^83,87^ It is important to note that a similar behavioural pattern has been identified among people who have suffered traumatic experiences, particularly sexual trauma, with a higher risk of engaging in risky sexual behaviours such as compulsive sexual behaviour and unprotected sexual intercourse.^116,117^ Considering that sexual traumatic history is greatly overrepresented among schizophrenia samples,^118^ future research should focus on exploring whether the presence of traumatic history could be a major mediating factor in this population.

Our findings pose significant implications for the understanding and care of individuals living with schizophrenia. It is essential to note that most of the studies included in our analyses involved samples that had undergone STI screening for research purposes. This hardly reflects the clinical reality of many centres, where routine screening is not commonly performed in patients with severe mental disorders. Tailored sex education and preventive measures (including regular screening for STIs) are essential for all members of society, and people with schizophrenia are no exception. Interventions targeted at individuals with severe mental health disorders must be put in place to reduce the burden associated with STIs and other adverse consequences of risky sexual behaviours.

### Limitations

The findings of this study should be interpreted considering certain limitations, primarily the significant heterogeneity detected in most of the studied outcomes. Although high heterogeneity is expected in proportional meta-analyses,^119^ samples included in this work were heterogeneous in terms of their geographic origin, severity and characteristics, which on the other hand allows for better generalisation of our results. Owing to a lack of data, some potentially moderating factors such as religion,^120^ antipsychotic treatment^121^ or access to sexual health services^122^ were not analysed. Furthermore, it was not possible to stratify the studied outcomes by sex, even though significant gender-related differences may exist.^123^ Another crucial determinant for the transmission of the infections studied is the use of injectable drugs. Although we addressed the effect of a comorbid substance use disorder on the prevalence of STIs through meta-regressions, unfortunately there were insufficient data to stratify the effect of each substance, or the injection route. In the case of sexual behaviour outcomes, most of the data in the original studies were obtained through self-report, which can be potentially subject to social desirability bias; this has proven to be particularly problematic in studies on this topic.^124^ Although it remains unclear whether this bias differentially affects populations with severe mental health disorders, it should be considered in future research. Finally, most of the studies included in this analysis were cross-sectional in nature. Longitudinal research is needed to better understand the temporal dynamics of sexual behaviour and STI risk in individuals with schizophrenia.

### Future implications

Patients with schizophrenia exhibit a high prevalence of STIs, having several-fold increased risks of HIV, HBV and HCV infection compared with the general population. Although individuals in this population are significantly less likely to be in a stable relationship or engage in sexual activity, they show extremely high prevalence of risky sexual behaviours, engaging in unprotected sexual relationships. These findings highlight the need to incorporate sexual health into the overall care framework for patients with schizophrenia, with the aim of preventing and treating sexually transmitted diseases.

## Supporting information

Aymerich et al. supplementary material Aymerich et al. supplementary material

## Data Availability

The data that support the findings of this study are available from the corresponding author, C.A., on reasonable request.

## References

[1] World Health Organization. *Sexual and Reproductive Health and Research*. WHO, n.d. (https://www.who.int/teams/sexual-and-reproductive-health-and-research-(srh)/overview).

[2] van Lankveld J, Jacobs N, Thewissen V, Dewitte M, Verboon P. The associations of intimacy and sexuality in daily life: temporal dynamics and gender effects within romantic relationships. J Soc Pers Relat 2018; 35: 557–76. 29899585 10.1177/0265407517743076 PMC5987853

[3] Fusar-Poli P, Estradé A, Stanghellini G, Venables J, Onwumere J, Messas G, et al. The lived experience of psychosis: a bottom-up review co-written by experts by experience and academics. World Psychiatry 2022; 21: 168–88. 35524616 10.1002/wps.20959 PMC9077608

[4] de Jager J, McCann E. Psychosis as a barrier to the expression of sexuality and intimacy: an environmental risk? Schizophr Bull 2017; 43: 236–9. 28049759 10.1093/schbul/sbw172 PMC5782497

[5] Yang J, Yu K, Wang X, Wang Y, Zhang C, Ma R, et al. Sexual needs of people with schizophrenia: a descriptive phenomenological study. BMC Psychiatry 2023; 23: 147. 36894926 10.1186/s12888-023-04640-z PMC9996993

[6] Kelly DL, Conley RR. Sexuality and schizophrenia: a review. Schizophr Bull 2004; 30: 767–79. 15954189 10.1093/oxfordjournals.schbul.a007130

[7] Higgins A, Barker P, Begley CM. Sexual health education for people with mental health problems: what can we learn from the literature? J Psychiatr Ment Health Nurs 2006; 13: 687–97. 17087671 10.1111/j.1365-2850.2006.01016.x

[8] Posada Correa AM, Andrade Carrillo RA, Suarez Vega DC, Gómez Cano S, Agudelo Arango LG, Tabares Builes LF, et al. Sexual and reproductive health in patients with schizophrenia and bipolar disorder. Rev Colomb Psiquiatr 2020; 49: 15–22. 10.1016/j.rcp.2018.04.007 32081203

[9] Khalifeh H, Oram S, Osborn D, Howard LM, Johnson S. Recent physical and sexual violence against adults with severe mental illness: a systematic review and meta-analysis. Int Rev Psychiatry 2016; 28: 433–51. 27645197 10.1080/09540261.2016.1223608 PMC5309869

[10] Liang C, Bai Y, Hsu J, Huang K, Ko N, Chu H, et al. The risk of sexually transmitted infections following first-episode schizophrenia among adolescents and young adults: a cohort study of 220 545 subjects. Schizophr Bull 2020; 46: 795–803. 32060532 10.1093/schbul/sbz126 PMC7344918

[11] Ciocca G, Jannini TB, Ribolsi M, Rossi R, Niolu C, Siracusano A, et al. Sexuality in ultra-high risk for psychosis and first-episode psychosis. A systematic review of literature. Front Psychiatry 2021; 12: 750033. 34777053 10.3389/fpsyt.2021.750033 PMC8579023

[12] Brüne M, Schaub D, Juckel G, Langdon R. Social skills and behavioral problems in schizophrenia: the role of mental state attribution, neurocognition and clinical symptomatology. Psychiatry Res 2011; 190: 9–17. 20417974 10.1016/j.psychres.2010.03.015

[13] Thornicroft G, Brohan E, Rose D, Sartorius N, Leese M. Global pattern of experienced and anticipated discrimination against people with schizophrenia: a cross-sectional survey. Lancet 2009; 373: 408–15. 19162314 10.1016/S0140-6736(08)61817-6

[14] Khokhar JY, Dwiel LL, Henricks AM, Doucette WT, Green AI. The link between schizophrenia and substance use disorder: a unifying hypothesis. Schizophr Res 2018; 194: 78–85. 28416205 10.1016/j.schres.2017.04.016 PMC6094954

[15] Lu C, Jin D, Palmer N, Fox K, Kohane IS, Smoller JW, et al. Large-scale real-world data analysis identifies comorbidity patterns in schizophrenia. Trans Psychiatry 2022; 12: 154. 10.1038/s41398-022-01916-y PMC9001711 35410453

[16] Cheng J, Hu J, Chang M, Lin M, Ku H, Chien R, et al. Hepatitis C–associated late-onset schizophrenia: a nationwide, population-based cohort study. J Psychiatry Neurosci 2021; 46: E583–91. 34728558 10.1503/jpn.200154 PMC8565883

[17] Fellerhoff B, Laumbacher B, Mueller N, Gu S, Wank R. Associations between *Chlamydophila* infections, schizophrenia and risk of HLA-A10. Mol Psychiatry 2007; 12: 264–72. 17102800 10.1038/sj.mp.4001925

[18] Dragioti E, Radua J, Solmi M, Arango C, Oliver D, Cortese S, et al. Global population attributable fraction of potentially modifiable risk factors for mental disorders: a meta-umbrella systematic review. Mol Psychiatry 2022; 27: 3510–9. 35484237 10.1038/s41380-022-01586-8 PMC9708560

[19] Radua J, Ramella-Cravaro V, Ioannidis JPA, Reichenberg A, Phiphopthatsanee N, Amir T, et al. What causes psychosis? An umbrella review of risk and protective factors. World Psychiatry 2018; 17: 49–66. 29352556 10.1002/wps.20490 PMC5775150

[20] Cournos F, McKinnon K, Sullivan G. Schizophrenia and comorbid human immunodeficiency virus or hepatitis C virus. J Clin Psychiatry 2005; 66(Suppl 6): 27–33. 16107181

[21] Page MJ, McKenzie JE, Bossuyt PM, Boutron I, Hoffmann TC, Mulrow CD, et al. The PRISMA 2020 statement: an updated guideline for reporting systematic reviews. BMJ 2021; 372: n71. 33782057 10.1136/bmj.n71 PMC8005924

[22] Stroup DF, Berlin JA, Morton SC, Olkin I, Williamson GD, Rennie D, et al. Meta-analysis Of Observational Studies in Epidemiology: a proposal for reporting. Meta-analysis of observational studies in epidemiology (MOOSE) group. JAMA 2000; 283: 2008–12. 10789670 10.1001/jama.283.15.2008

[23] Altman DG, Simera I, Hoey J, Moher D, Schulz K. EQUATOR: reporting guidelines for health research. Lancet 2008; 371: 1149–50. 18395566 10.1016/S0140-6736(08)60505-X

[24] American Psychiatric Association. Diagnostic and Statistical Manual of Mental Disorders. APA, 2022.

[25] World Health Organization. *International Classification of Diseases, Eleventh Revision (ICD-11)*. WHO, 2021.

[26] Wells GA, Shea B, O'Connell D, Peterson J, Welch V, Losos M, et al. *The Newcastle-Ottawa Scale (NOS) for assessing the quality of nonrandomised studies in meta-analyses*. Ottawa Hospital Research Institute, 2012 (https://www.ohri.ca/programs/clinical_epidemiology/oxford.asp).

[27] Ioannidis JPA, Patsopoulos NA, Evangelou E. Uncertainty in heterogeneity estimates in meta-analyses. BMJ 2007; 335: 914. 17974687 10.1136/bmj.39343.408449.80 PMC2048840

[28] R Core Team. R: a language and environment for statistical computing. R Foundation for Statistical Computing, 2021.

[29] Viechtbauer W. Package ‘Metafor’. The Comprehensive R Archive Network, 2015.

[30] Mbewe E, Haworth A, Welham J, Mubanga D, Chazulwa R, Zulu MM, et al. Clinical and demographic features of treated first-episode psychotic disorders: a Zambian study. Schizophr Res 2006; 86: 202. 16765568 10.1016/j.schres.2006.03.046

[31] Opondo PR, Ho-Foster AR, Ayugi J, Hatitchki B, Pumar M, Bilker WB, et al. HIV prevalence among hospitalized patients at the main psychiatric referral hospital in Botswana. AIDS Behav 2018; 22: 1503–16. 28831617 10.1007/s10461-017-1878-3 PMC6348889

[32] Mwelase MP, Ntlantsana V, Tomita A, Chiliza B, Paruk S. HIV prevalence and access to HIV testing and care in patients with psychosis in South Africa. S Afr J Psychiatr 2023; 29: 1918. 36756542 10.4102/sajpsychiatry.v29i0.1918 PMC9900311

[33] Lundberg P, Nakasujja N, Musisi S, Thorson AE, Cantor-Graae E, Allebeck P. HIV prevalence in persons with severe mental illness in Uganda: a cross-sectional hospital-based study. Int J Ment Health Syst 2013; 7: 20. 23866085 10.1186/1752-4458-7-20 PMC3724693

[34] Maling S, Todd J, Van der Paal L, Grosskurth H, Kinyanda E. HIV-1 seroprevalence and risk factors for HIV infection among first-time psychiatric admissions in Uganda. AIDS Care 2011; 23: 171. 21259129 10.1080/09540121.2010.498939

[35] Mona K, Ntlantsana V, Tomita AM, Paruk S. Prevalence of cannabis use in people with psychosis in KwaZulu-natal, South Africa. S Afr J Psychiatr 2022; 28: 1927. 36340643 10.4102/sajpsychiatry.v28i0.1927 PMC9634825

[36] Doufik J, Zemmama H, Bouri S, Rabhi S, Boujraf S, Aalouane R, et al. Prevalence of sexually transmitted infections in patients with schizophrenia in Morocco. Infect Dis Now 2022; 52: 304. 35248765 10.1016/j.idnow.2022.02.010

[37] Gonzalez-Torres MA, Salazar MA, Inchausti L, Ibañez B, Pastor J, Gonzalez G, et al. Lifetime sexual behavior of psychiatric inpatients. J Sex Med 2010; 7: 3045. 20367769 10.1111/j.1743-6109.2010.01795.x

[38] Hart DJ, Heath RG, Sautter FJ, Schwartz BD, Garry RF, Choi B, et al. Antiretroviral antibodies: implications for schizophrenia, schizophrenia spectrum disorders, and bipolar disorder. Biol Psychiatry 1999; 45: 704. 10188000 10.1016/s0006-3223(98)00229-7

[39] Blank MB, Mandell DS, Aiken L, Hadley TR. Co-occurrence of HIV and serious mental illness among medicaid recipients. Psychiatr Serv 2002; 53: 868. 12096171 10.1176/appi.ps.53.7.868

[40] Rosenberg SD, Drake RE, Brunette MF, Wolford GL, Marsh BJ. Hepatitis C virus and HIV co-infection in people with severe mental illness and substance use disorders. AIDS 2005; 19(Suppl 3): S26–33. 16251824 10.1097/01.aids.0000192067.94033.aa

[41] Closson K, McLinden T, Patterson TL, Eyawo O, Kibel M, Card KG, et al. HIV, schizophrenia, and all-cause mortality: a population-based cohort study of individuals accessing universal medical care from 1998 to 2012 in British Columbia, Canada. Schizophr Res 2019; 209: 198–205. 31255392 10.1016/j.schres.2019.04.020

[42] Baillargeon JG, Paar DP, Wu H, Giordano TP, Murray O, Raimer BG, et al. Psychiatric disorders, HIV infection and HIV/hepatitis co-infection in the correctional setting. AIDS Care 2008; 20: 124. 18278623 10.1080/09540120701426532

[43] Rodgers-Johnson PE, Hickling FW, Irons A, Johnson BK, Irons-Morgan M, Stone GA, et al. Retroviruses and schizophrenia in Jamaica. Mol Chem Neuropathol 1996; 28: 237. 8871965 10.1007/BF02815228

[44] Prince JD, Walkup J, Akincigil A, Amin S, Crystal S. Serious mental illness and risk of New HIV/AIDS diagnoses: an analysis of medicaid beneficiaries in eight states. Psychiatr Serv 2012; 63: 1032. 22855268 10.1176/appi.ps.201100342

[45] Himelhoch S, Mccarthy JF, Ganoczy D, Medoff D, Kilbourne A, Goldberg R, et al. Understanding associations between serious mental illness and hepatitis C virus among veterans: a national multivariate analysis. Psychosomatics 2009; 50: 30. 19213970 10.1176/appi.psy.50.1.30 PMC3774160

[46] Doyle ME, Labbate LA. Incidence of HIV infection among patients with new-onset psychosis. Psychiatr Serv 1997; 48: 237. 9021857 10.1176/ps.48.2.237

[47] Walkup J, Akincigil A, Amin S, Hoover D, Siegel M, Crystal S. Prevalence of diagnosed HIV disease among medicaid beneficiaries with schizophrenia in U.S. Metropolitan areas. J Nerv Ment Dis 2010; 198: 682. 20823732 10.1097/NMD.0b013e3181ef21a2

[48] Santos da Silva AS, Santos Costa FJL, Câmara JT, Das Neves FM, De Assis JT. Disease prevalence in infectious care center of users of psychosocial caxias-MA [Prevalência de doenças infecciosas em usuários de centro de atenção psicossocial de caxias-MA]. Revista de Pesquisa 2018; 10: 137.

[49] Brown E, Castagnini E, Langstone A, Mifsud N, Gao C, McGorry P, et al. High-risk sexual behaviours in young people experiencing a first episode of psychosis. Early Interv Psychiatry 2023; 17: 159. 35355426 10.1111/eip.13301

[50] Hariri AG, Karadag F, Gokalp P, Essizoglu A. Risky sexual behavior among patients in Turkey with bipolar disorder, schizophrenia, and heroin addiction. J Sex Med 2011; 8: 2284. 21492406 10.1111/j.1743-6109.2011.02282.x

[51] Williams J, Barclay M, Omana C, Buten S, Post JJ. Universal blood-borne virus screening in patients with severe mental illness managed in an outpatient clozapine clinic: uptake and prevalence. Australas Psychiatry 2020; 28: 186. 32019350 10.1177/1039856220901464

[52] Jallow A, Ljunggren G, Wändell P, Wahlström L, Carlsson AC. HIV-infection and psychiatric illnesses - a double edged sword that threatens the vision of a contained epidemic: the greater Stockholm HIV cohort study. J Infect 2017; 74: 22. 27717780 10.1016/j.jinf.2016.09.009

[53] Grassi L, Pavanati M, Cardelli R, Ferri S, Peron L. HIV-risk behaviour and knowledge about HIV/AIDS among patients with schizophrenia. Psychol Med 1999; 29: 171. 10077305 10.1017/s0033291798007818

[54] Bauer-Staeb C, Jörgensen L, Lewis G, Dalman C, Osborn DPJ, Hayes JF. Prevalence and risk factors for HIV, hepatitis B, and hepatitis C in people with severe mental illness: a total population study of Sweden. Lancet Psychiatry 2017; 4: 685. 28687481 10.1016/S2215-0366(17)30253-5 PMC5573766

[55] De Hert M, Franic T, Vidovic D, Wampers M, Van Eyck D, Van Herck K, et al. Prevalence of HIV and hepatitis C infection among patients with schizophrenia. Schizophr Res 2009; 108: 307. 19091513 10.1016/j.schres.2008.11.008

[56] Heslin M, Jewell A, Croxford S, Chau C, Smith S, Pittrof R, et al. Prevalence of HIV in mental health service users: a retrospective cohort study. BMJ Open 2023; 13: e067337. 10.1136/bmjopen-2022-067337 PMC10186409 37185201

[57] Karabulut N. The frequency of hepatitis B virus, hepatitis C virus and human immunodeficiency virus infections among patients with schizophrenia in a mental health hospital in Turkey. Viral Hepat J 2016; 22: 48–51.

[58] Sockalingam S, Shammi C, Powell V, Barker L, Remington G. Determining rates of hepatitis C in a clozapine treated cohort. Schizophr Res 2010; 124: 86–90. 20605572 10.1016/j.schres.2010.06.005

[59] Freudenreich O, Gandhi RT, Walsh JP, Henderson DC, Goff DC. Hepatitis C in schizophrenia: screening experience in a community-dwelling clozapine cohort. Psychosomatics 2007; 48: 405. 17878499 10.1176/appi.psy.48.5.405

[60] Chang C, Liu C, Chen S, Tsai H. Hepatitis C virus and hepatitis B virus in patients with schizophrenia. Medicine (Baltimore) 2021; 100: e26218. 34087899 10.1097/MD.0000000000026218 PMC8183751

[61] Nakamura Y, Koh M, Miyoshi E, Ida O, Morikawa M, Tokuyama A, et al. High prevalence of the hepatitis C virus infection among the inpatients of schizophrenia and psychoactive substance abuse in Japan. Prog Neuropsychopharmacol Biol Psychiatry 2004; 28: 591. 15093967 10.1016/j.pnpbp.2004.01.018

[62] Chiu Y, Lin H, Kuo N, Kao S, Lee H. Increased risk of concurrent hepatitis C among male patients with schizophrenia. Psychiatry Res 2017; 258: 217. 28844561 10.1016/j.psychres.2017.08.036

[63] Carney CP, Jones L, Woolson RF. Medical comorbidity in women and men with schizophrenia: a population-based controlled study. J Gen Intern Med 2006; 21: 1133. 17026726 10.1111/j.1525-1497.2006.00563.x PMC1831667

[64] Hung C, Loh E, Hu T, Chiu H, Hsieh H, Chan C, et al. Prevalence of hepatitis B and hepatitis C in patients with chronic schizophrenia living in institutions. J Chin Med Assoc 2012; 75: 275. 22721622 10.1016/j.jcma.2012.03.002

[65] Cuadrado A, Cabezas J, Llerena S, Nieves Salceda JF, Fortea JI, Crespo-Facorro B, et al. Prevalence of hepatitis C in patients with non-affective psychotic disorders. Rev Esp Enferm Dig 2020; 112: 550. 32579015 10.17235/reed.2020.7278/2020

[66] Fuller BE, Rodriguez VL, Linke A, Sikirica M, Dirani R, Hauser P. Prevalence of liver disease in veterans with bipolar disorder or schizophrenia. Gen Hosp Psychiatry 2011; 33: 232. 21601719 10.1016/j.genhosppsych.2011.03.006

[67] Huckans MS, Blackwell AD, Harms TA, Hauser P. Management of hepatitis C disease among VA patients with schizophrenia and substance use disorders. Psychiatr Serv 2006; 57: 403. 16525001 10.1176/appi.ps.57.3.403

[68] Zhu H, Liu X, Xue Y, Shen C, Li Y, Wang A, et al. Seroepidemiology of hepatitis B virus infection among Chinese schizophrenia patients. J Infect Dev Ctries 2015; 9: 512. 25989171 10.3855/jidc.5416

[69] Wang Y, Yu L, Zhou H, Zhou Z, Zhu H, Li Y, et al. Serologic and molecular characteristics of hepatitis B virus infection in vaccinated schizophrenia patients in China. J Infect Dev Ctries 2016; 10: 427. 27131009 10.3855/jidc.7377

[70] Imani M, Sharafi H, Sadeh A, Kakavand-Ghalehnoei R, Alavian SM, Fotouhi A. Seroprevalence of hepatitis B virus and hepatitis C virus infections among people with severe mental illness in Tehran, Iran. Hepat Mon 2022; 22: e126696.

[71] Alvarado Esquivel C, Arreola Valenzuela MA, Mercado Suárez MF, Espinoza-Andrade F. Hepatitis B virus infection among inpatients of a psychiatric hospital of Mexico. Clin Pract Epidemiol Ment Health 2005; 1: 10. 16053524 10.1186/1745-0179-1-10 PMC1198240

[72] Said WM, Saleh R, Jumaian N. Prevalence of hepatitis B virus among chronic schizophrenia patients. East Mediterr Health J 2001; 7: 526. 12690775

[73] Krause D, Matz J, Weidinger E, Wagner J, Wildenauer A, Obermeier M, et al. The association of infectious agents and schizophrenia. World J Biol Psychiatry 2010; 11: 739. 20602604 10.3109/15622971003653246

[74] Zhang Q, Xie J. Association between schizophrenia and syphilis: a retrospective study in xiamen, China. BMC Psychiatry 2018; 18: 273. 30176838 10.1186/s12888-018-1869-6 PMC6122212

[75] Chaudhury S, Chandra S, Augustine M. Prevalence of Australia antigen (HBsAg) in institutionalised patients with psychosis. Br J Psychiatry 1994; 164: 542. 8038945 10.1192/bjp.164.4.542

[76] Bianco CL, Pratt SI, Ferron JC. Deficits in sexual interest among adults with schizophrenia: another look at an old problem. Psychiatr Serv 2019; 70: 1000. 31401908 10.1176/appi.ps.201800403

[77] Ma M, Chao J, Hung J, Sung S, Chao IC. Sexual activity, sexual dysfunction, and sexual life quality among psychiatric hospital inpatients with schizophrenia. J Sex Med 2018; 15: 324. 29502981 10.1016/j.jsxm.2018.01.008

[78] Acuña MJ, Martín JC, Graciani M, Cruces A, Gotor F. A comparative study of the sexual function of institutionalized patients with schizophrenia. J Sex Med 2010; 7: 3414. 20456629 10.1111/j.1743-6109.2010.01832.x

[79] Fortier P, Mottard J, Trudel G, Even S. Study of sexuality-related characteristics in young adults with schizophrenia treated with novel neuroleptics and in a comparison group of young adults. Schizophr Bull 2003; 29: 559. 14609249 10.1093/oxfordjournals.schbul.a007028

[80] Raja M, Azzoni A. Sexual behavior and sexual problems among patients with severe chronic psychoses. Eur Psychiatry 2003; 18: 70. 12711402 10.1016/s0924-9338(03)00009-9

[81] McCann E. The expression of sexuality in people with psychosis: breaking the taboos. J Adv Nurs 2000; 32: 132. 10886444 10.1046/j.1365-2648.2000.01452.x

[82] Kazour F, Obeid S, Hallit S. Sexual desire and emotional reactivity in chronically hospitalized Lebanese patients with schizophrenia. Perspect Psychiatr Care 2020; 56: 502. 31750549 10.1111/ppc.12455

[83] Cournos F, Guido JR, Coomaraswamy S, Meyer-Bahlburg H, Sugden R, Horwath E. Sexual activity and risk of HIV infection among patients with schizophrenia. Am J Psychiatry 1994; 151: 228. 8296894 10.1176/ajp.151.2.228

[84] Hannachi N, El Kissi Y, Samoud S, Nakhli J, Letaief L, Gaabout S, et al. High prevalence of human herpesvirus 8 in schizophrenic patients. Psychiatry Res 2014; 216: 192. 24560611 10.1016/j.psychres.2013.12.035

[85] Mccann E. The sexual and relationship needs of people who experience psychosis: quantitative findings of a UK study. J Psychiatr Ment Health Nurs 2010; 17: 295–303. 20529179 10.1111/j.1365-2850.2009.01522.x

[86] Brown A, Lubman DI, Paxton SJ. Reducing sexually-transmitted infection risk in young people with first-episode psychosis. Int J Ment Health Nurs 2011; 20: 12–20. 21199240 10.1111/j.1447-0349.2010.00700.x

[87] Negash B, Asmamewu B, Alemu WG. Risky sexual behaviors of schizophrenic patients: a single center study in Ethiopia, 2018. BMC Res Notes 2019; 12: 635. 31558159 10.1186/s13104-019-4673-6 PMC6764125

[88] Klaf FS. Female homosexuality and paranoid schizophrenia. Arch Gen Psychiatry 1961; 4: 84. 13756539 10.1001/archpsyc.1961.01710070086011

[89] Lindamer LA, Buse DC, Auslander L, Unützer J, Bartels SJ, Jeste DV. A comparison of gynecological variables and service Use among older women with and without schizophrenia. Psychiatr Serv 2003; 54: 902. 12773608 10.1176/appi.ps.54.6.902

[90] Simiyon M, Chandra PS, Desai G. Sexual dysfunction among women with schizophrenia – a cross sectional study from India. Asian J Psychiatr 2016; 24: 93. 27931918 10.1016/j.ajp.2016.08.022

[91] Shaikh RAK, Ghogare AS, Prasad P, Deshmukh S. A cross-sectional study of antipsychotic drugs induced sexual dysfunction among married males with remitted schizophrenia attending tertiary health care centre from central India. J Clin Diagn Res 2021; 15: VC01–7.

[92] Miller LJ, Finnerty M. Sexuality, pregnancy, and childrearing among women with schizophrenia-spectrum disorders. Psychiatr Serv 1996; 47: 502. 8740491 10.1176/ps.47.5.502

[93] Bai Y, Huang Y, Lin C, Chen J. Emerging homosexual conduct during hospitalization among chronic schizophrenia patients. Acta Psychiatr Scand 2000; 102: 350. 11098806 10.1034/j.1600-0447.2000.102005350.x

[94] Carey MP, Carey KB, Maisto SA, Gordon CM, Vanable PA. Prevalence and correlates of sexual activity and HIV-related risk behavior among psychiatric outpatients. J Consult Clin Psychol 2001; 69: 846. 11680563 10.1037//0022-006x.69.5.846 PMC2424203

[95] Wright ER, Wright DE, Perry BL, Foote-Ardah CE. Stigma and the sexual isolation of people with serious mental illness. Soc Probl 2007; 54: 78–98.

[96] Ancedere A, Kucuk L. Sexual life and associated factors in psychiatric patients. Sexuality and Disability 2017; 35: 89–106.

[97] Carey MP, Carey KB, Maisto SA, Gleason JR, Gordon CM, Brewer KK. HIV-risk behavior among outpatients at a state psychiatric hospital: prevalence and risk modeling. Behav Ther 1999; 30: 389–406. 19626130 10.1016/S0005-7894(99)80017-3 PMC2713728

[98] Mclennan JD, Ganguli R. Family planning and parenthood needs of women with severe mental illness: clinicians’ perspective. Community Ment Health J 1999; 35: 369. 10452703 10.1023/a:1018770109042

[99] Miller LJ, Finnerty M. Family planning knowledge, attitudes and practices in women with schizophrenic spectrum disorders. J Psychosom Obstet Gynaecol 1998; 19: 210. 9929847 10.3109/01674829809025699

[100] Özcan NK, Boyacıoğlu NE, Enginkaya S, Dinç H, Bilgin H. Reproductive health in women with serious mental illnesses. J Clin Nurs 2014; 23: 1283. 24720577 10.1111/jocn.12332

[101] Tozoglu E, Aydin N, Yalcin S, Kasali K. Unintended and unwanted pregnancies in women with major psychiatric disorders: a cross-sectional comparative study. Psychiatry Clin Psychopharmacol 2020; 30: 1.

[102] Lluch E, Miller BJ. Rates of hepatitis B and C in patients with schizophrenia: a meta-analysis. Gen Hosp Psychiatry 2019; 61: 41. 31710857 10.1016/j.genhosppsych.2019.10.007

[103] Chen S, Chiang J, Hsu C, Shen Y. Schizophrenia is associated with an increased risk of sexually transmitted infections: a nationwide population-based cohort study in Taiwan. Schizophr Res 2018; 202: 316. 29954703 10.1016/j.schres.2018.06.050

[104] Gebeyehu DA, Mulatie M. Risky sexual behavior and its associated factors among patients with severe mental disorder in university of gondar comprehensive specialized hospital, 2018. BMC Psychiatry 2021; 21: 51. 33478422 10.1186/s12888-021-03054-z PMC7818773

[105] Chhagan U, Ntlantsana V, Tomita A, Chiliza B, Paruk S. The dual burden of HIV infection and first-episode psychosis in Africa: a systematic review and meta-analysis. J Nerv Ment Dis 2021; 209: 600. 34397760 10.1097/NMD.0000000000001366

[106] Wu B, Tobe RG, Yan M, Lin H, Zhou H. Trends of global burden related to HBV and HCV from 1990 to 2019: an age–period–cohort analysis. J Med Virol 2023; 95: e28663. 36905287 10.1002/jmv.28663

[107] Pawlotsky J, Negro F, Aghemo A, Berenguer M, Dalgard O, Dusheiko G, et al. EASL recommendations on treatment of hepatitis C 2018. J Hepatol 2018; 69: 461–511. 29650333 10.1016/j.jhep.2018.03.026

[108] Smith JM, Uvin AZ, Macmadu A, Rich JD. Epidemiology and treatment of hepatitis B in prisoners. Curr Hepatol Rep 2017; 16: 178–83. 29450123 10.1007/s11901-017-0364-8 PMC5808981

[109] Prins SJ. Prevalence of mental illnesses in U.S. State prisons: a systematic review. Psychiatr Serv 2014; 65: 862. 24686574 10.1176/appi.ps.201300166 PMC4182175

[110] Hutchinson G, Bhugra D, Mallett R, Burnett R, Corridan B, Leff J. Fertility and marital rates in first-onset schizophrenia. Soc Psychiatry Psychiatr Epidemiol 1999; 34: 617. 10703270 10.1007/s001270050183

[111] Blom JD, Mangoenkarso E. Sexual hallucinations in schizophrenia spectrum disorders and their relation with childhood trauma. Front Psychiatry 2018; 9: 193. 29867612 10.3389/fpsyt.2018.00193 PMC5954108

[112] Park YW, Kim Y, Lee JH. Antipsychotic-Induced sexual dysfunction and its management. World J Mens Health 2012; 30: 153. 23596605 10.5534/wjmh.2012.30.3.153 PMC3623530

[113] Kolotkin RL, Zunker C, Østbye T. Sexual functioning and obesity: a review. Obesity 2012; 20: 2325. 22522887 10.1038/oby.2012.104

[114] Korchia T, Achour V, Faugere M, Albeash A, Yon DK, Boyer L, et al. Sexual dysfunction in schizophrenia a systematic review and meta-analysis. JAMA Psychiatry 2023; 80(11): 1110–20. 37703012 10.1001/jamapsychiatry.2023.2696 PMC10500435

[115] Li X, Wu J, Liu J, Li K, Wang F, Sun X, et al. The influence of marital status on the social dysfunction of schizophrenia patients in community. Int J Nurs Sci 2015; 2: 149.

[116] Werner KB, Cunningham-Williams RM, Sewell W, Agrawal A, McCutcheon VV, Waldron M, et al. The impact of traumatic experiences on risky sexual behaviors in black and white young adult women. Womens Health Issues 2018; 28: 421. 29903544 10.1016/j.whi.2018.04.011 PMC6143429

[117] Slavin MN, Scoglio AAJ, Blycker GR, Potenza MN, Kraus SW. Child sexual abuse and compulsive sexual behavior: a systematic literature review. Curr Addict Rep 2020; 7: 76–88. 33425653 10.1007/s40429-020-00298-9 PMC7787260

[118] Schäfer I, Fisher HL. Childhood trauma and psychosis – what is the evidence? Dialogues Clin Neurosci 2011; 13: 360. 22033827 10.31887/DCNS.2011.13.2/ischaefer PMC3182006

[119] Barker TH, Migliavaca CB, Stein C, Colpani V, Falavigna M, Aromataris E, et al. Conducting proportional meta-analysis in different types of systematic reviews: a guide for synthesisers of evidence. BMC Med Res Methodol 2021; 21: 1–189. 34544368 10.1186/s12874-021-01381-z PMC8451728

[120] McFarland MJ, Uecker JE, Regnerus MD. The role of religion in shaping sexual frequency and satisfaction: evidence from married and unmarried older adults. J Sex Res 2011; 48: 297–308. 20349390 10.1080/00224491003739993 PMC3119480

[121] de Boer MK, Castelein S, Wiersma D, Schoevers RA, Knegtering H. The facts about sexual (Dys)function in schizophrenia: an overview of clinically relevant findings. Schizophr Bull 2015; 41: 674. 25721311 10.1093/schbul/sbv001 PMC4393701

[122] Johnson BT, Scott-Sheldon LAJ, Huedo-Medina TB, Carey MP. Interventions to reduce sexual risk for human immunodeficiency virus in adolescents: a meta-analysis of trials, 1985–2008. JAMA Pediatrics 2011; 165: 77–84. 10.1001/archpediatrics.2010.251 PMC4361805 21199984

[123] Thara R, Srinivasan TN. Marriage and gender in schizophrenia. Indian J Psychiatry 1997; 39: 64. 21584047 PMC2967086

[124] King BM. The influence of social desirability on sexual behavior surveys: a review. Arch Sex Behav 2022; 51: 1495–501. 35142972 10.1007/s10508-021-02197-0 PMC8917098

