# Supplementary material for: Sexually transmitted infections, sexual life and risk behaviours of people living with schizophrenia: systematic review and meta-analysis
Source: BJPsych Open. 2024 May 10;10(3):e110. doi: 10.1192/bjo.2024.49 (PMC11094452; doi:10.1192/bjo.2024.49)
Supplement: Aymerich et al. supplementary material [file S2056472424000498sup001.docx]

**SUPPLEMENTARY MATERIAL**

**Table S1:** PRISMA statement and checklist………………………………………………………………………………………................page 2-3

**Table S2**: MOOSE Statement checklist………………………..…………………………………………………………………………….page 4-5

**Table S3**: Search terms for the systematic review…………..………………………………………………………………………………….page 6

**Table S4**: Studied outcomes for sexual behavior…………..………………………..………………………………………………………….page 7

**Table S5**: Quality assessment: Newcastle-Ottawa Scale (NOS) for Cohort Studies……………………………………………………...…....page 8

**Figure S1**: PRISMA 2020 Flow Diagrams………………………..………………………………………………………………..…..……..page 9

**Table S6**: Characteristics of the included studies for the Sexual Behaviours Systematic Review and Meta-Analysis………..……….…page 10-11

**Figure S2:** Forest plots……………………………………………..……………………………………………………………….……..page 12-16

**Table S7:** Meta-regressions for the studied sexually transmitted infections………..………………………………………………….……..page 17

**Table S8:** Meta-regressions for the studied sex outcomes………..………………………………………………………………….…...…..page 18

**Table S9:** Subgroup analyses for the studied sexually transmitted infections……………………………….…………..…………….……..page 19

**Table S10:** Subgroup analyses for the studied sex outcomes.………..……………………………………………………...………………..page 20

**Figure S3:** Funnel plots for publication bias.………..…………………………………………………………………….……….……..page 21-23

**References………………………………….**.………..…………………………………………………………………….……….……..page 24-27

**Table S1:** PRISMA statement and checklist.

| **Section/Topic** | **Item #** | **Checklist item** | **Page** |
| --- | --- | --- | --- |
| **TITLE** | | | |
| Title | 1 | Identify the report as a systematic review. | 1 |
| **ABSTRACT** |  |  |  |
| Abstract | 2 | Provide a structured summary including, as applicable: background; objectives; data sources; study eligibility criteria, participants, and interventions; study appraisal and synthesis methods; results; limitations; conclusions and implications of key findings; systematic review registration number. | 3-4 |
| **INTRODUCTION** | | | |
| Rationale | 3 | Describe the rationale for the review in the context of existing knowledge. | 5 |
| Objectives | 4 | Provide an explicit statement of the objective(s) or question(s) the review addresses. | 5 |
| **METHODS** | | | |
| Eligibility criteria | 5 | Specify the inclusion and exclusion criteria for the review and how studies were grouped for the syntheses. | 6 |
| Information sources | 6 | Specify all databases, registers, websites, organisations, reference lists and other sources searched or consulted to identify studies. Specify the date when each source was last searched or consulted. | 6, eMS1 |
| Search strategy | 7 | Present the full search strategies for all databases, registers and websites, including any filters and limits used. | S3 |
| Selection process | 8 | Specify the methods used to decide whether a study met the inclusion criteria of the review, including how many reviewers screened each record and each report retrieved, whether they worked independently, and if applicable, details of automation tools used in the process. | 6, eMS1 |
| Data collection process | 9 | Specify the methods used to collect data from reports, including how many reviewers collected data from each report, whether they worked independently, any processes for obtaining or confirming data from study investigators, and if applicable, details of automation tools used in the process. | 6 |
| Data items | 10 | List and define all outcomes for which data were sought and if any assumptions were made about any missing or unclear information. | 6, S4 |
| Study risk of bias assessment | 11 | Specify the methods used to assess risk of bias in the included studies, including details of the tool(s) used, how many reviewers assessed each study and whether they worked independently, and if applicable, details of automation tools used in the process. | 7 |
| Effect measures | 12 | Specify for each outcome the effect measure(s) (e.g. risk ratio, mean difference) used in the synthesis or presentation of results. | 7-8 |
| Synthesis methods | 13 | Describe the processes used to decide which studies were eligible for each synthesis. Describe any methods required to prepare the data for presentation or synthesis, such as handling of missing summary statistics, or data conversions. Describe any methods used to tabulate or visually display results of individual studies and syntheses. Describe any methods used to synthesize results and provide a rationale for the choice(s). Describe the model(s), method(s) to identify the presence and extent of statistical heterogeneity, and software package(s) used, any methods used to explore possible causes of heterogeneity among study results (e.g. subgroup analysis, meta-regression) and any sensitivity analyses conducted to assess robustness of the synthesized results. | 6-8 |
| Reporting bias assessment | 14 | Describe any methods used to assess risk of bias due to missing results in a synthesis (arising from reporting biases). | 8 |
| Certainty assessment | 15 | Describe any methods used to assess certainty (or confidence) in the body of evidence for an outcome. | 7 |
| **RESULTS** | | | |
| Study selection | 16 | Describe the results of the search and selection process, from the number of records identified in the search to the number of studies included in the review, ideally using a flow diagram. Cite studies that might appear to meet the inclusion criteria, but which were excluded, and explain why they were excluded. | 8, 11 |
| Study characteristics | 17 | Cite each included study and present its characteristics. | 15-17, S6 |
| Risk of bias in studies | 18 | Present assessments of risk of bias for each included study. | 15-17, S6 |
| Results of individual studies | 19 | For all outcomes, present, for each study: (a) summary statistics for each group (where appropriate) and (b) an effect estimate and its precision (e.g. confidence/credible interval), ideally using structured tables or plots. | 19-22, S2 |
| Results of syntheses | 20 | For each synthesis, briefly summarise the characteristics and risk of bias among contributing studies. Present results of all statistical syntheses conducted. If meta-analysis was done, present for each the summary estimate and its precision (e.g. confidence/credible interval) and measures of statistical heterogeneity. If comparing groups, describe the direction of the effect. Present results of all investigations of possible causes of heterogeneity among study results and all sensitivity analyses conducted to assess the robustness of the synthesized results. | 15-17, S6 |
| Reporting biases | 21 | Present assessments of risk of bias due to missing results (arising from reporting biases) for each synthesis assessed. | 8-11, S2 |
| Certainty of evidence | 22 | Present assessments of certainty (or confidence) in the body of evidence for each outcome assessed. | 15-17, S6 |
| **DISCUSSION** | | | |
| Discussion | 23 | Provide a general interpretation of the results in the context of other evidence. Discuss any limitations of the evidence included in the review, any limitations of the review processes used and the implications of the results for practice, policy, and future research. | 12-14 |
| **OTHER INFORMATION** | | | |
| Registration and protocol | 24 | Provide registration information for the review, including register name and registration number, or state that the review was not registered. Indicate where the review protocol can be accessed, or state that a protocol was not prepared. Describe and explain any amendments to information provided at registration or in the protocol. | 6 |
| Support | 25 | Describe sources of financial or non-financial support for the review, and the role of the funders or sponsors in the review. | 1 |
| Competing interests | 26 | Declare any competing interests of review authors. | 1 |
| Availability of data, code and other materials | 27 | Report which of the following are publicly available and where they can be found: template data collection forms; data extracted from included studies; data used for all analyses; analytic code; any other materials used in the review. | Supps |

**Table S2:** MOOSE Statement - Reporting Checklist for Authors, Editors, and Reviewers of Meta-analyses of Observational Studies

| **Reporting Criteria** | **Reported (Yes/No)** | **Reported on Page** |
| --- | --- | --- |
| **Reporting of Background** |  |  |
| Problem definition | Yes | 5 |
| Hypothesis statement | Yes | 5 |
| Description of Study Outcome(s) | Yes | 5, 7 |
| Type of exposure or intervention used | Yes | 6 |
| Type of study design used | Yes | 1 |
| Study population | Yes | 6 |
| **Reporting of Search Strategy** |  |  |
| Qualifications of searchers (eg, librarians and investigators) | Yes | 6 |
| Search strategy, including time period included in the synthesis and keywords | Yes | 6, S4 |
| Effort to include all available studies, including contact with authors | Yes | 6, eMS1 |
| Databases and registries searched | Yes | eMS1 |
| Search software used, name and version, including special features used (eg, explosion) | N.a. | N.a. |
| Use of hand searching (eg, reference lists of obtained articles) | Yes | 6 |
| List of citations located and those excluded, including justification | Yes | S1 |
| Method for addressing articles published in languages other than English | Yes | 6 |
| Method of handling abstracts and unpublished studies | Yes | 6 |
| Description of any contact with authors | N.a. | N.a. |
| **Reporting of Methods** |  |  |
| Description of relevance or appropriateness of studies assembled for assessing the hypothesis to be tested | Yes | 5 |
| Rationale for the selection and coding of data (eg, sound clinical principles or convenience) | Yes | 6,7 |
| Documentation of how data were classified and coded (eg, multiple raters, blinding, and interrater reliability) | Yes | 6 |
| Assessment of confounding (eg, comparability of cases and controls in studies where appropriate) | Yes | 6 |
| Assessment of study quality, including blinding of quality assessors; stratification or regression on possible predictors of study results YES 5 | Yes | 7 |
| Assessment of heterogeneity | Yes | 7 |
| Description of statistical methods (eg, complete description of fixed or random effects models, justification of whether  the chosen models account for predictors of study results, dose-response models, or cumulative meta-analysis) in sufficient detail to be replicated | Yes | 7 |
| Provision of appropriate tables and graphics | Yes | 15-21, Supps |
| **Reporting of Results** |  |  |
| Table giving descriptive information for each study included | Yes | 15-17, S6 |
| Results of sensitivity testing (eg, subgroup analysis) | Yes | S9, S10 |
| Indication of statistical uncertainty of findings | Yes | 15-17, S6 |
| **Reporting of Discussion** |  |  |
| Quantitative assessment of bias (eg, publication bias) | Yes | 8-11, S2 |
| Justification for exclusion (eg, exclusion of non–English-language citations) | Yes | 6 |
| Assessment of quality of included studies | Yes | 15-17, S6 |
| **Reporting of Conclusions** |  |  |
| Consideration of alternative explanations for observed results | Yes | 12 - 14 |
| Generalization of the conclusions (ie, appropriate for the data presented and within the domain of the literature review) | Yes | 13, 14 |
| Guidelines for future research | Yes | 14 |
| Disclosure of funding source | Yes | 1 |

**Table S3:** Search terms for the systematic review.

| **Sexual behavior** |
| --- |
| (TS=("schizophrenia" OR "psychosis" OR "schizoaffective" OR "non-affective psychosis" OR "psychotic")) AND TS=("sexuality" OR "birth control" OR "sexual behavior" OR "sexual behaviour" OR "sex behavior" OR "sexual behaviour" OR "sex habits" OR "sexual habits" OR "sex behavior" OR "sex behaviour" OR "heterosexual*" OR "homosexual*" OR "intercourse" OR "contracepti*" OR "condom*" OR "family planning*" OR “sexual orientation”) |

| **Sexually transmitted diseases** |
| --- |
| ("syphilis" OR "treponema" OR "hepatitis" OR "hepatitis B" OR "hepatitis C" OR "HBV" OR "HCV" OR "human papillomavirus" OR "Papillomaviridae" OR "hpv" OR "Chlamydia" OR "trachomatis" OR "gonorrh*" OR "Mycoplasma genitalium" OR "Human immunodeficiency virus" OR "HIV" OR "acquired immunodeficiency syndrome" OR "AIDS") AND ("schizophrenia" OR "psychosis" OR "schizoaffective" OR "non-affective psychosis" OR "psychotic") |

**Table S4:** Studied outcomes for sexual behavior.

| **Outcome** | **Description** |
| --- | --- |
| Sexually transmitted infection | An infection that results from transmission of a pathogenic organism by sexual contact and that accounts for a noticeable amount of illness in the general population. |
| % Stable relationship | Proportion of patients self-defining to be in a stable romantic relationship. |
| % Lifetime sexual relationships | Proportion of patients who declare having had sexual relationships with another individual at least once in their lifetime. |
| % Satisfaction with sex life | Proportion of patients who declared to be satisfied with their sex life (if continuous measure, scores equal or greater than 5/10 or equivalent were included). |
| % Interest in sexual relationships | Proportion of patients who declared to be interested in maintaining sexual relationships with other people. |
| % Sexually active | Proportion of patients who declare to be sexually active (defined of having had at least one sexual relationship with another individual in the previous 12 months). |
| % Prostitution use | Proportion of patients declaring to have given money or other goods in exchange for sexual relationships. |
| % Prostitution work | Proportion of patients declaring to have received money or other goods in exchange for sexual relationships. |
| % Consistent use of condom | Proportion of patients declaring to have a consistent use of condoms in their sexual relationships (if categorical measure, “always” and “almost always” were included under this section). |
| % Hormonal contraception | Proportion of patients declaring to be users of hormonal contraception (themselves or their partners). |
| % Unprotected sexual relationships | Proportion of patients declaring to maintain sexual relationships with any method to prevent unplanned pregnancies or sexually transmitted diseases. |
| % Unplanned pregnancy | Proportion of patients declaring to have had an unplanned pregnancy (themselves or their partners). |
| % Multiple partners | Proportion of patients declaring to have more than one sexual partner during the same period of time. |
| Age at first sexual relationships | Mean (± standard deviation) age of patients at the time of their first sexual relationship. |

**Table S5:** Quality assessment: Newcastle-Ottawa Scale (NOS) for Cohort Studies

Quality of the included studies was assessed using the Newcastle-Ottawa Scale (NOS) for Cohort Studies due to the heterogeneity expected in the included studies. The following assessment scores were used:

| **Criteria** | **Maximum Score** |
| --- | --- |
| Representativeness of exposed cohort | ★ |
| Selection of the non-exposed cohort | ★ |
| Ascertainment of exposure | ★ |
| Demonstration that outcome of interest was not present at start of study | ★ |
| Comparability of cohorts based on the design or analysis controlled for confounders | ★ ★ |
| Assessment of outcome | ★ |
| Was follow-up long enough for outcomes to occur | ★ |
| Adequacy of follow-up of cohorts | ★ |

**Figure S1.** PRISMA 2020 Flow Diagram for Sexually Transmitted Diseases Systematic Review and Meta-Analysis (S1-A) and PRISMA 2020 Flow Diagram for Sexual Behaviours Systematic Review and Meta-Analysis (S1-B) and.


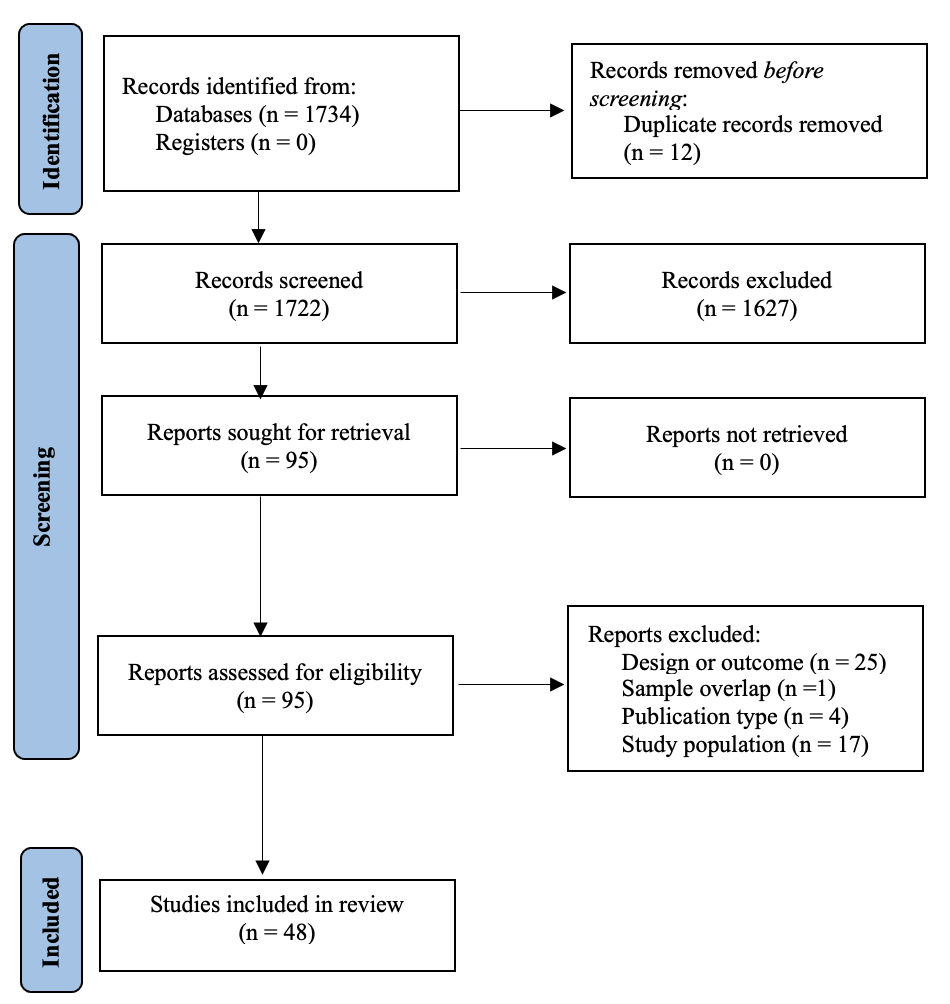

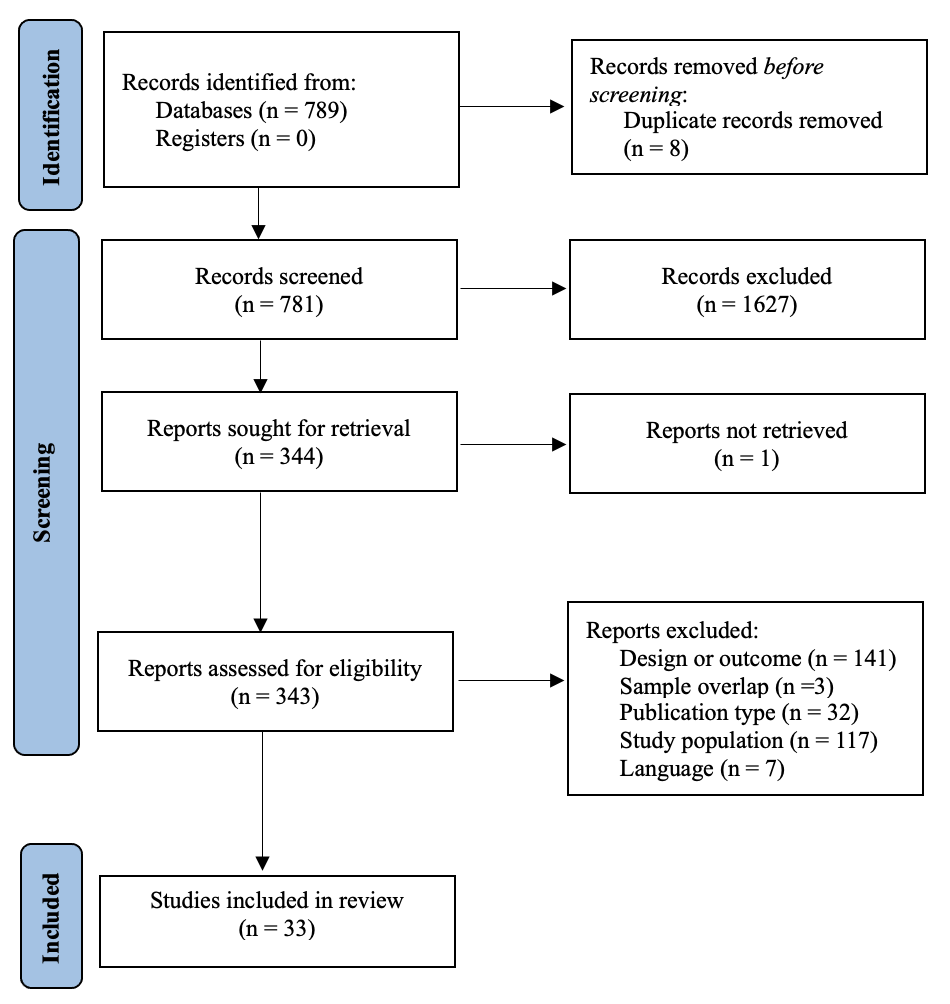


**A**

**B**

**Table S6:** Characteristics of the included studies for the Sexual Behaviours Systematic Review and Meta-Analysis.

| **Author year** | **Country** | **N** | **Age mean (SD)** | **% Women** | **Setting** | **% Stable relationship** | **Outcome(s) of interest** | **NOS** |
| --- | --- | --- | --- | --- | --- | --- | --- | --- |
| Franklin et al, 1961 ^1^ | United Kingdom | 75 | 35.2 (N.a.) | 100% | Inpatient | 49% | % Stable relationship | 3 |
| Brown et al, 2011-A ^2^ | Australia | 67 | 22.0 (2.4) | 28% | Outpatient | 43% | % Consistent use of condom | 8 |
| Jager et al, 2018 ^3^ | Netherlands | 28 | 42.0 (10.2) | 32% | Outpatient | 39% | % Stable relationship, % Interest in SSRR | 7 |
| Incedere et al, 2017 ^4^ | Turkey | 50 | N.a. | 58% | Outpatient | N.a. | % Sexually active, % Consistent use of condom, % Hormonal contraceptive use, % Multiple partners | 8 |
| Simiyon et al, 2016 ^5^ | India | 63 | 33.3 (4.3) | 100% | Outpatient | 100% | % Stable relationship | 7 |
| Ma et al, 2018 ^6^ | Taiwan | 317 | 47.7 (9.5) | 25% | Inpatient | 8% | % Stable relationship, % Lifetime SSRR, % Sex life satisfaction, % Interest in SSRR, % Sexually active, Age at first SSRR | 8 |
| Hannachi et al, 2014 ^7^ | Tunisia | 108 | 36.8 (11.2) | 34% | Outpatient | 35% | % Stable relationship, % Sexually active, % Multiple partners | 7 |
| Carey et al, 1999 ^8^ | USA | 460 | N.a. | N.a. | Outpatient | N.a. | % Sexually active | 6 |
| Raja et al, 2003 ^9^ | Italy | 39 | 30.9 (6.9) | 31% | N.a. | 13% | % Stable relationship, % Sexually active, % Consistent use of condom, % Hormonal contraceptive use, % Unprotected SSRR | 7 |
| Grassi et al, 1999 ^10^ | Italy | 91 | 35.3 (8.1) | 35% | Outpatient | 15% | % Stable relationship, % Lifetime SSRR, % Sexually active, % Prostitution use, % Consistent use of condom, % Multiple partners | 7 |
| McLennan et al, 1999 ^11^ | USA | 268 | 46.1 (12.7) | 100% | N.a. | N.a. | % Sexually active, % Unprotected SSRR | 5 |
| Bai et al, 2000 ^12^ | Taiwan | 113 | 36.2 (6.2) | 51% | Inpatient | N.a. | % Lifetime SSRR, Age at first SSRR | 5 |
| Carey et al, 2001 ^13^ | USA | 296 | N.a. | N.a. | Outpatient | N.a. | % Sexually active | 6 |
| McCann et al, 2000 ^14^ | United Kingdom | 11 | N.a. | 36% | Inpatient | 18% | % Lifetime SSRR, % Sexually active | 5 |
| Fortier et al, 2003 | Canada | 45 | 24.4 (4.9) | 44% | Outpatient | 9% | % Stable relationship, % Lifetime SSRR, % Sexually active | 7 |
| Carey et al, 2004 ^15^ | USA | 146 | N.a. | N.a. | Outpatient | N.a. | % Sexually active | 6 |
| Brown et al, 2022 ^16^ | Australia | 69 | 19.6 (2.8) | 42% | Outpatient | 22% | % Stable relationship, % Lifetime SSRR, % Unprotected SSRR, % Unplanned pregnancy | 8 |
| McCann et al, 2010 ^17^ | United Kingdom | 30 | 40.9 (10.0) | 50% | Outpatient | 40% | % Stable relationship, % Sex life satisfaction, % Sexually active | 6 |
| Brown et al, 2011-B ^18^ | Australia | 67 | 22.0 (2.4) | 28% | Outpatient | 43% | % Stable relationship, Age at first SSRR | 8 |
| González-Torres et al, 2010 ^19^ | Spain | 235 | N.a. | N.a. | N.a. | N.a. | % Lifetime SSRR, % Consistent use of condom | 8 |
| Acuña et al, 2010 ^20^ | Spain | 116 | 40.9 (10.2) | 24% | Inpatient | 9% | % Stable relationship, Age at first SSRR | 7 |
| Hariri et al, 2011 ^21^ | Turkey | 88 | 34.9 (8.8) | 64% | N.a. | 49% | % Stable relationship, % Sexually active, % Prostitution use, % Prostitution use, % Unprotected SSRR, % Unplanned pregnancy, % Multiple partners | 6 |
| Ozcan et al, 2013 | Turkey | 55 | N.a. | 100% | N.a. | N.a. | % Unplanned pregnancy | 7 |
| Kazour et al, 2019 ^22^ | Lebanon | 60 | N.a. | 50% | Inpatient | 22% | % Stable relationship, % Sexually active | 7 |
| Cournos et al, 1994 ^23^ | USA | 95 | 36.0 (8.3) | 26% | N.a. | 26% | % Stable relationship, % Sexually active, % Prostitution use, % Consistent use of condom, % Multiple partners | 8 |
| Bianco et al, 2018 ^24^ | USA | 231 | 48.1 (12.7) | 48% | Outpatient | 7% | % Stable relationship, % Interest in SSRR | 8 |
| Lindamer et al, 2003 ^25^ | USA | 65 | 57.6 (6.1) | 100% | Outpatient | 83% | % Stable relationship, % Hormonal contraceptive use | 6 |
| Miller et al, 1996 ^26^ | USA | 44 | 30.8 (7.7) | 100% | Outpatient | N.a. | % Sex life satisfaction, % Sexually active, % Prostitution use, % Unplanned pregnancy, Age at first SSRR | 6 |
| Negash et al, 2019 ^27^ | Ethiopia | 429 | 32.8 (8.9) | 41% | Outpatient | 46% | % Stable relationship, % Prostitution use, % Unprotected SSRR, % Multiple partners | 9 |
| Wright et al, 2007 ^28^ | USA | 290 | N.a. | N.a. | N.a. | N.a. | % Sexually active | 6 |
| Miller et al, 1998 ^29^ | USA | 44 | 30.8 (7.7) | 100% | N.a. | N.a. | % Unprotected SSRR, Age at first SSRR | 7 |
| Ozcan et al, 2020 | Turkey | 30 | N.a. | 100% | N.a. | N.a. | % Unplanned pregnancy | 6 |
| Shaikh et al, 2021 ^30^ | India | 100 | 31.3 (6.1) | 0% | Outpatient | 100% | % Stable relationship | 6 |

STI Sexually transmitted disease, HC Healthy controls, SD Standard deviation, SUD Substance use disorder, NOS Newcastle-Ottawa Scale, HIV Human immunodeficiency virus, HBV Hepatitis B virus, HCV Hepatitis C virus, USA United States of America

**Figure S2:** Forest plots.

**
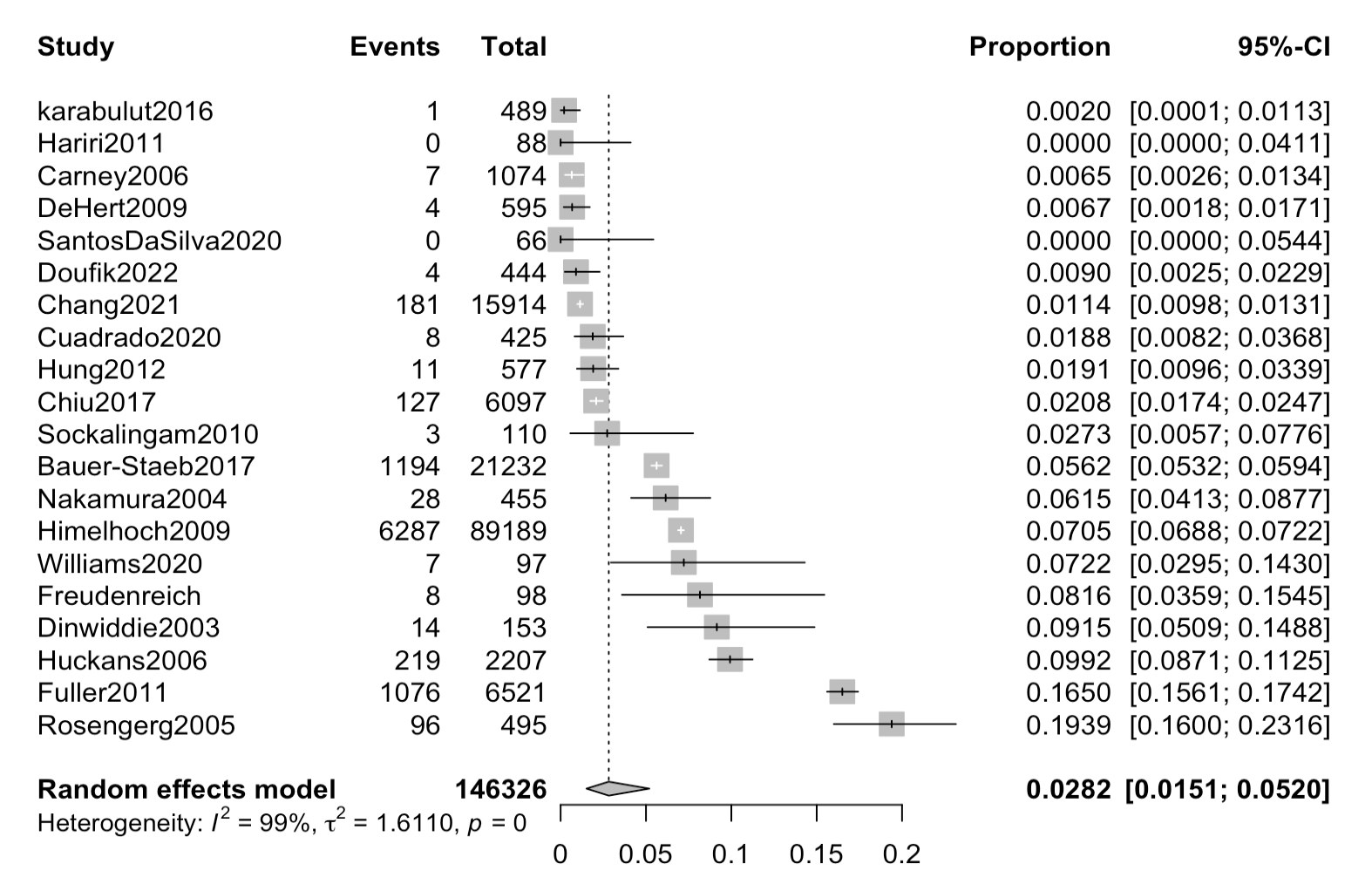
**

**Figure S2-A**. HCV Prevalence

**
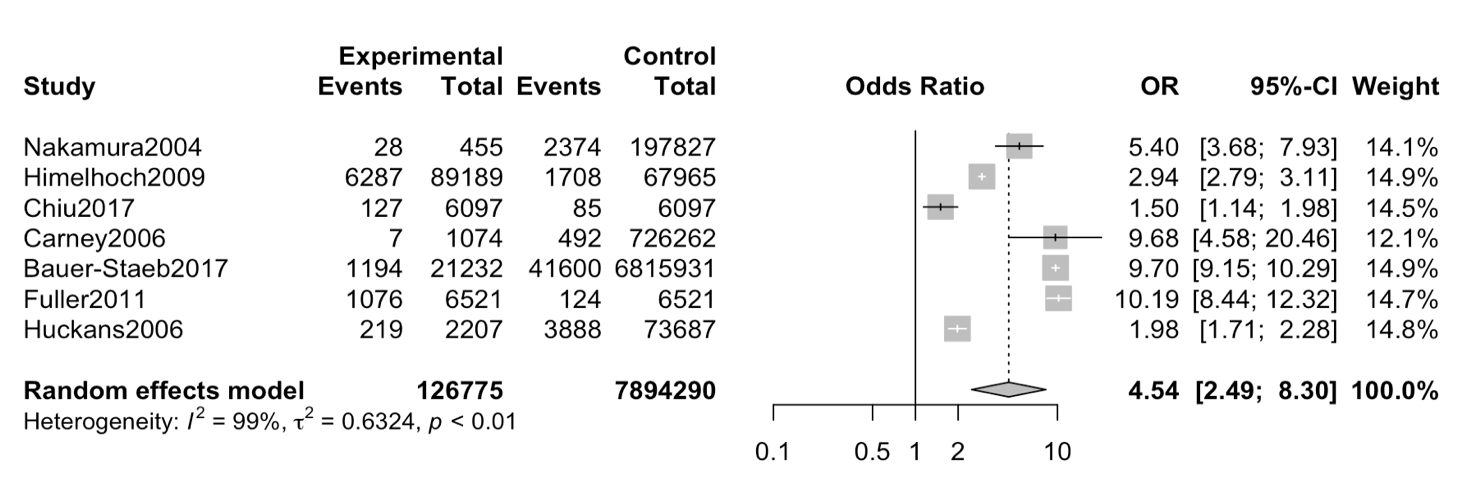
**

**Figure S2-B**. HCV Odds Ratio

**
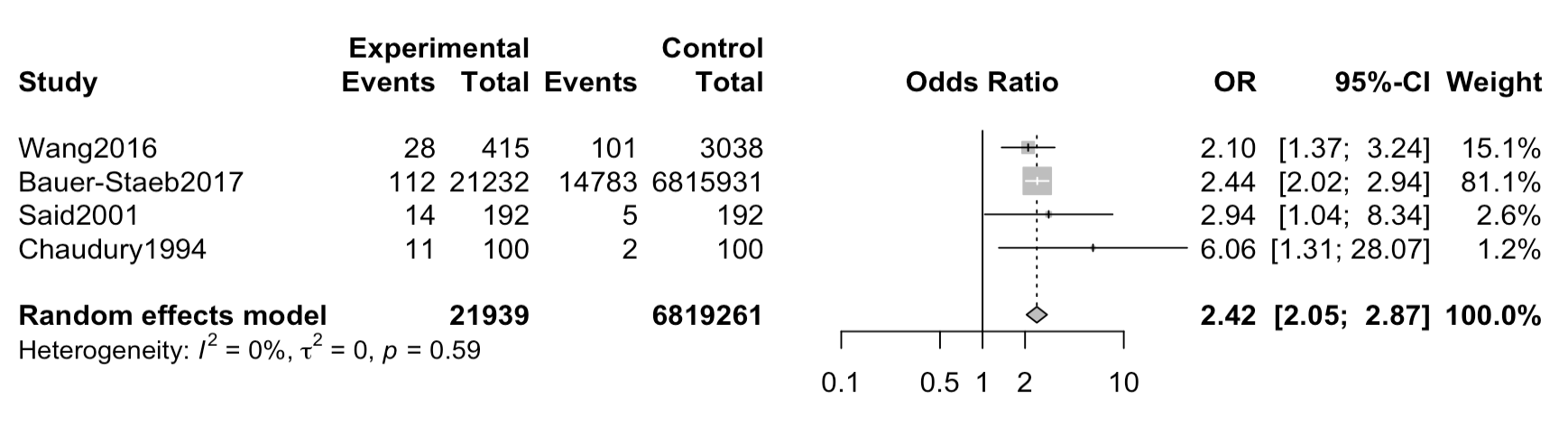

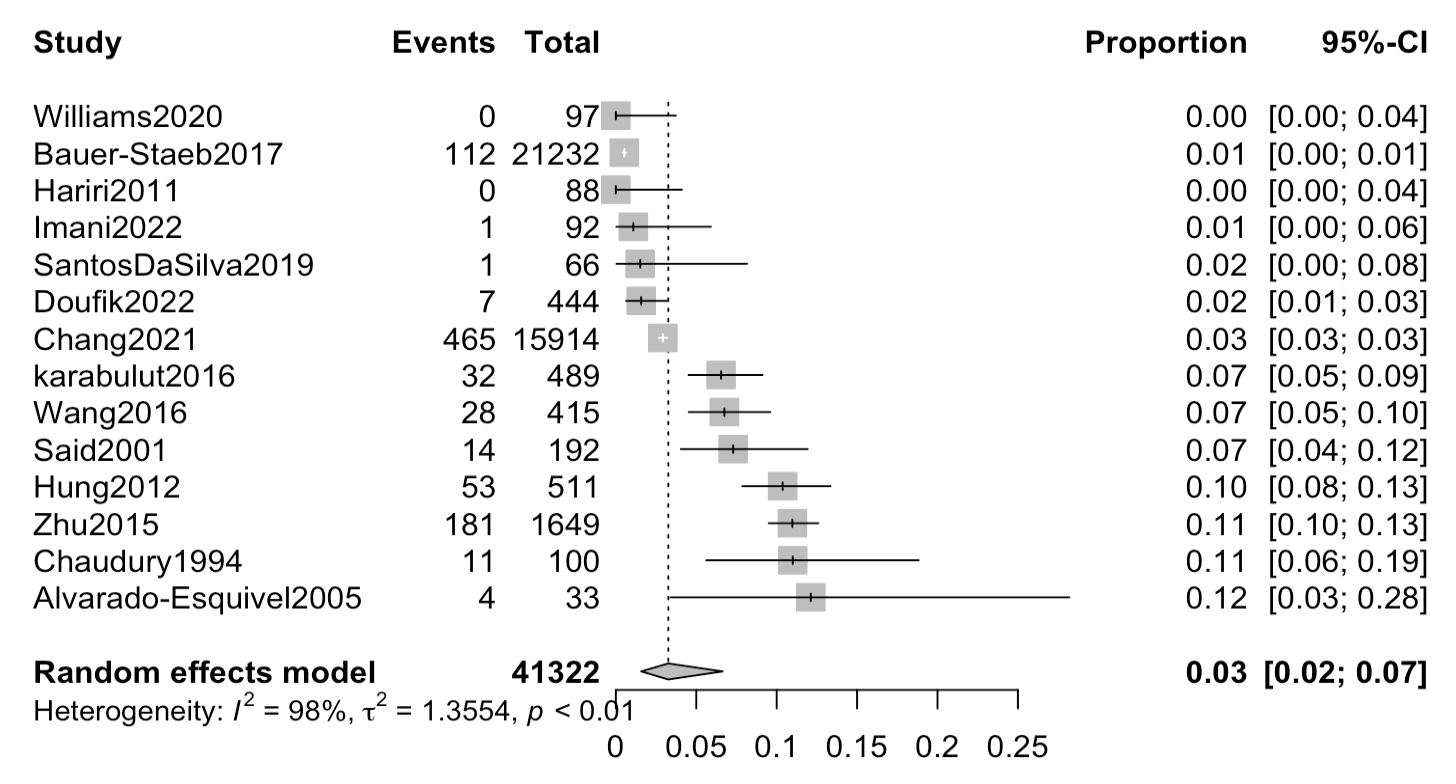
**

**Figure S2-D**. HBV Odds Ratio

**Figure S2-C**. HBV Prevalence


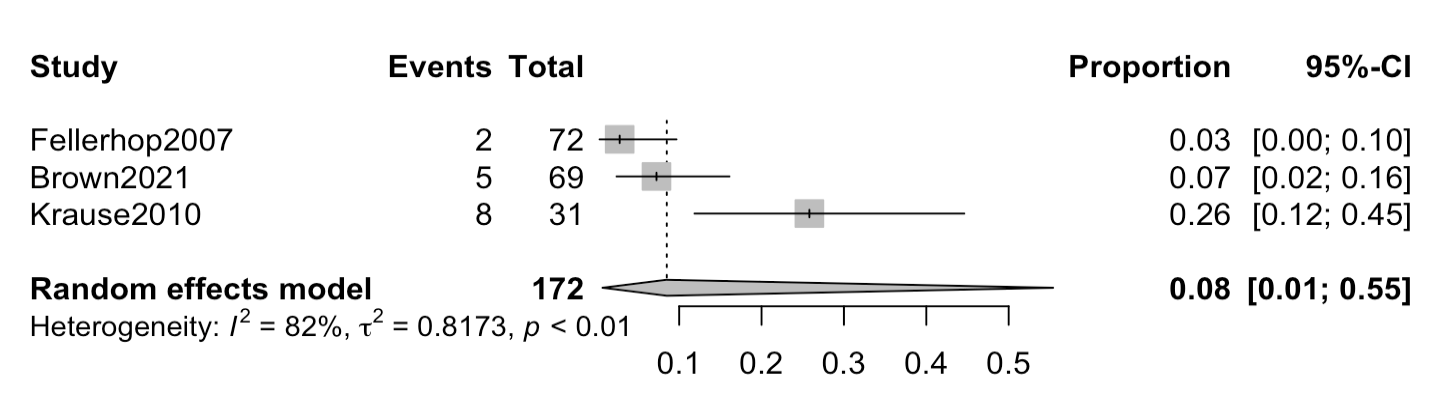
**
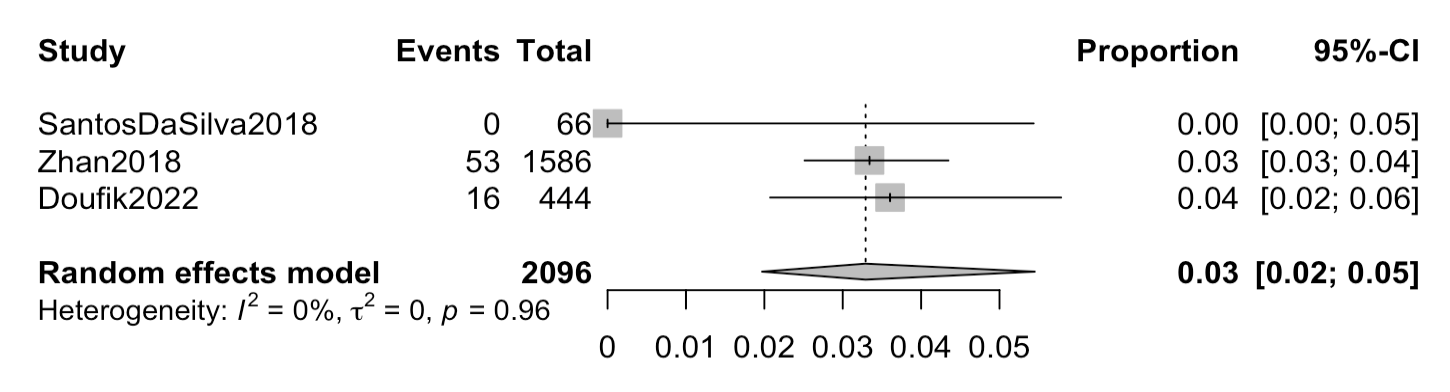
**

**Figure S2-E**. Chlamydia trachomatis Prevalence

**Figure S2-F**. Treponema pallidum Prevalence


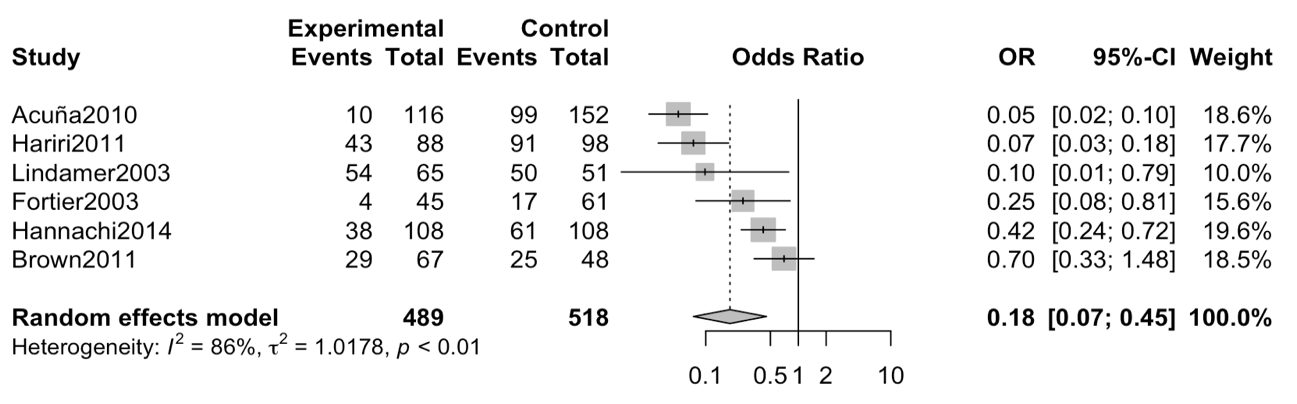

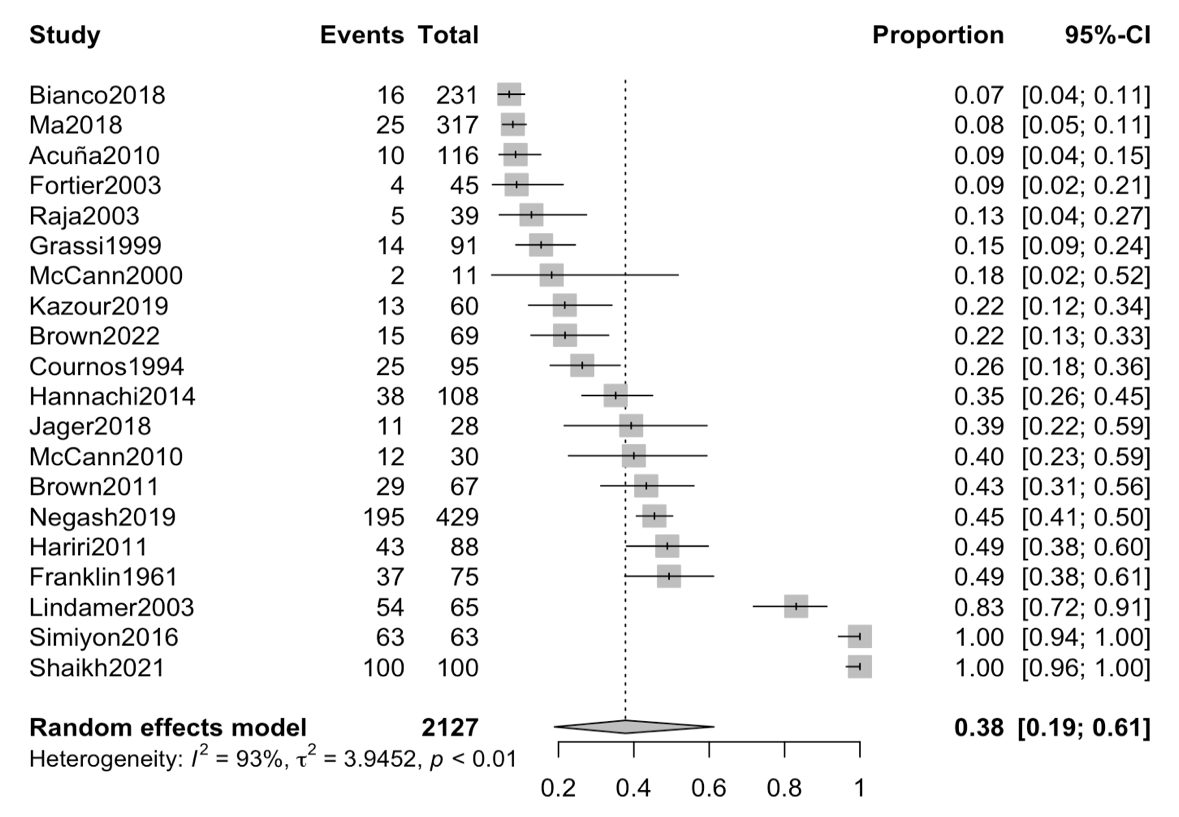


**Figure S2-H**. Odds ratio for being in a stable romantic relationship.

**Figure S2-G**. Proportion of patients in a stable romantic relationship.


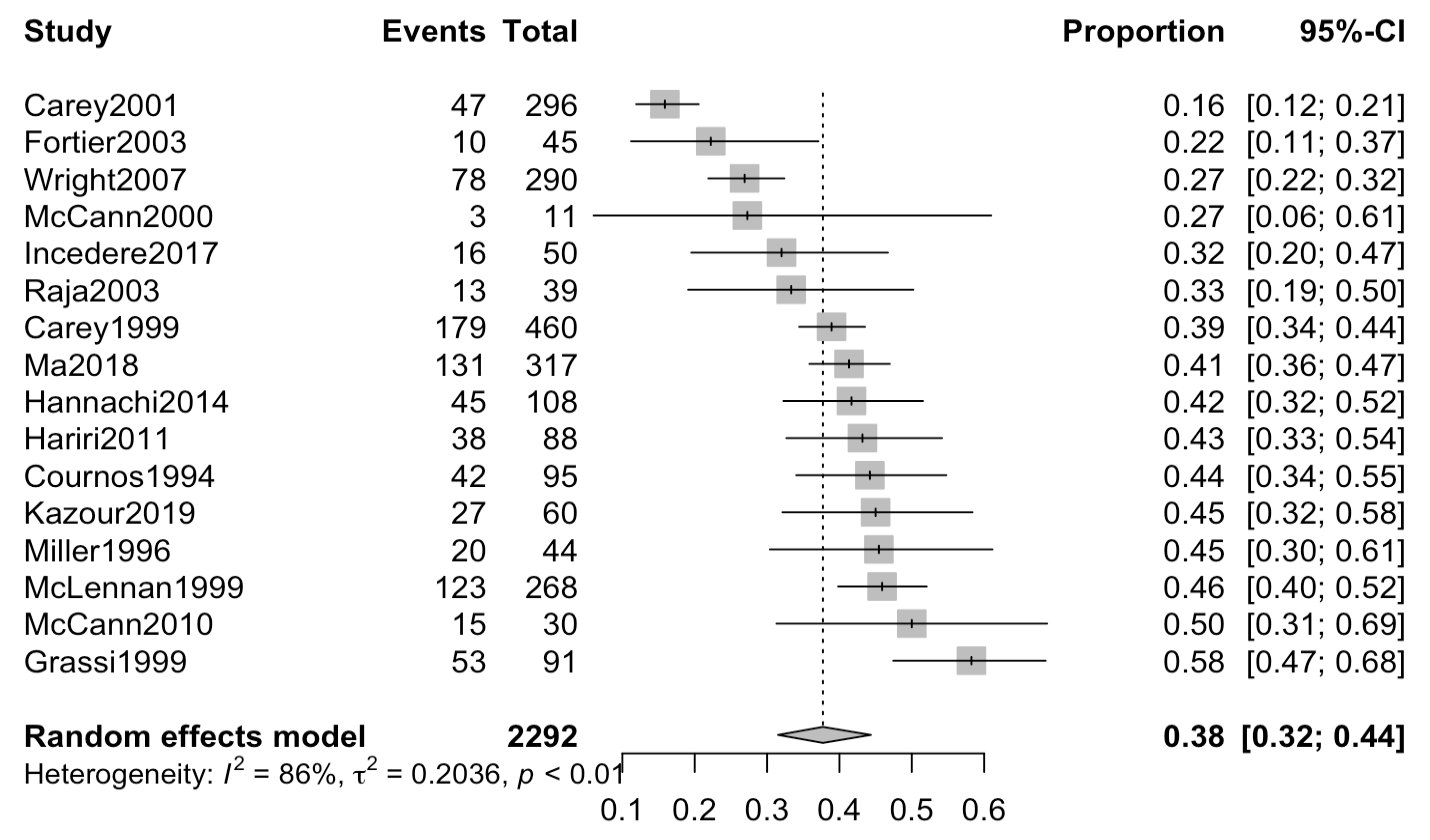


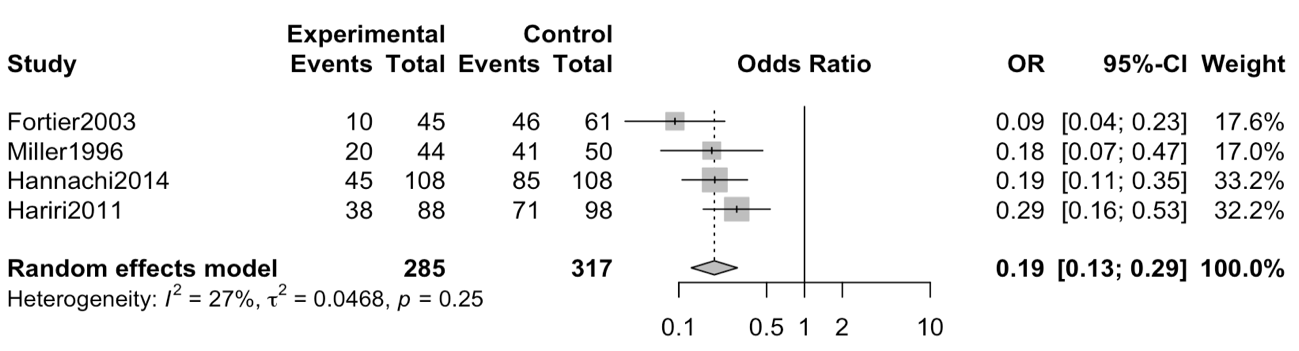


**Figure S2-J**. Odds ratio for being sexually active.

**Figure S2-I**. Proportion of sexually active patients.

**Table S7:** Meta-regressions for the studied sexually transmitted infections.

| **Meta-regression** | **No. of**  **Studies** | **β Coefficient** | **SE** | **95% CI** | | **P value** |
| --- | --- | --- | --- | --- | --- | --- |
| **HIV Prevalence** | | | | | | |
| **Publication year** | 28 | 0.010 | 0.044 | -0.080 | 0.099 | 0.83 |
| **Mean age** | 13 | -0.051 | 0.069 | -0.202 | 0.101 | 0.48 |
| **% Female** | 19 | 0.250 | 3.449 | -7.028 | 7.527 | 0.94 |
| **% SUD** | 8 | 8.079 | 1.659 | 0.003 | 4.020 | <0.01* |
| **% Stable relationship** | 11 | -1.596 | 3.880 | -10.373 | -1.082 | 0.69 |
| **NOS Scale** | 28 | 0.219 | 0.284 | -0.366 | 0.802 | 0.45 |
| **HCV Prevalence** | | | | | | |
| **Publication year** | 20 | -0.097 | 0.043 | -0.187 | -0.007 | 0.04* |
| **Mean age** | 14 | 0.143 | 0.024 | 0.090 | 0.196 | <0.01* |
| **% Female** | 14 | -3.222 | 2.127 | -7.856 | 1.411 | 0.16 |
| **% SUD** | 5 | 4.201 | 1.103 | 0.692 | 7.710 | 0.03* |
| **NOS Scale** | 20 | -0.210 | 0.315 | -0.871 | 0.451 | 0.51 |
| **HBV Prevalence** | | | | | | |
| **Publication year** | 14 | -0.082 | 0.034 | -0.157 | -0.007 | 0.03* |
| **Mean age** | 10 | 0.008 | 0.033 | -0.069 | 0.084 | 0.82 |
| **% Female** | 9 | -0.747 | 1.858 | -5.139 | 3.646 | 0.70 |
| **NOS Scale** | 14 | -0.233 | 0.243 | -0.763 | 0.297 | 0.36 |
| **HIV Odds Ratio** | | | | | | |
| **Publication year** | 7 | 0.036 | 0.037 | -0.060 | 0.131 | 0.38 |
| **NOS Scale** | 7 | 0.002 | 0.267 | -0.683 | 0.687 | 0.99 |
| **HCV Odds Ratio** | | | | | | |
| **Publication year** | 7 | -0.008 | 0.068 | -0.183 | 0.168 | 0.92 |
| **NOS Scale** | 7 | 0.625 | 0.277 | -0.088 | 1.338 | 0.07 |

SE Standard Error; CI Confidence Interval; NOS Newcastle-Ottawa Scale; SUD Substance Use Disorder; HIV Human Immunodeficiency Virus; HBV Hepatitis B Virus; HCV Hepatitis C Virus

**Table S8:** Meta-regressions for the studied sexual and risk behaviour outcomes.

| **Meta-regression** | **No. of**  **Studies** | **β Coefficient** | **SE** | **95% CI** | | **P value** |
| --- | --- | --- | --- | --- | --- | --- |
| **Proportion of schizophrenia patients in a stable romantic relationship** | | | | | | |
| **Publication year** | 20 | 0.016 | 0.033 | -0.054 | 0.086 | 0.64 |
| **Mean age** | 18 | -0.009 | 0.055 | -0.126 | 0.178 | 0.87 |
| **% Female** | 20 | 0.015 | 0.015 | -0.016 | 0.045 | 0.32 |
| **NOS Scale** | 20 | -0.383 | 0.340 | -1.096 | 0.331 | 0.27 |
| **Proportion of sexually active schizophrenia patients** | | | | | | |
| **Publication year** | 16 | 0.002 | 0.016 | -0.033 | 0.036 | 0.92 |
| **Mean age** | 10 | 0.017 | 0.015 | -0.017 | 0.053 | 0.28 |
| **% Female** | 13 | 0.002 | 0.002 | -0.004 | 0.007 | 0.51 |
| **% Stable relationship** | 10 | 0.699 | 0.851 | -1.264 | 2.662 | 0.44 |
| **NOS Scale** | 16 | 0.062 | 0.139 | -0.237 | 0.360 | 0.66 |
| **Proportion of schizophrenia patients with lifetime sexual relationships** | | | | | | |
| **Publication year** | 7 | -0.022 | 0.042 | -0.130 | 0.086 | 0.62 |
| **NOS Scale** | 7 | 0.218 | 0.299 | -0.550 | 0.985 | 0.50 |

SE Standard Error; CI Confidence Interval; NOS Newcastle-Ottawa Scale

**Table S9:** Subgroup analyses for the studied sexually transmitted infections.

|  | **No. Studies** | **Sample size** | **Proportion** | **95% CI** | **Heterogeneity** | |
| --- | --- | --- | --- | --- | --- | --- |
|  |  |  |  |  | **I^2^ (%)** | ***p*** |
| **HIV Prevalence** | | | | | | |
| **Continent** | Test for between groups difference: Q = 11.11; p = 0.049* | | | | | |
| North America | 10 | 2377675 | 0.0203 | 0.0098 – 0.0417 | 99.8 | 0.00 |
| Africa | 7 | 2124 | 0.0732 | 0.0151 – 0.2894 | 90.00 | 0.00 |
| Europe | 6 | 41583 | 0.0067 | 0.0020 – 0.0222 | 98.1 | 0.00 |
| Oceania | 2 | 166 | 0.0060 | 0.0000 – 0.9995 | N.a. | N.a. |
| Asia | 2 | 88 | 0.0000 | 0.0000 – 1.0000 | N.a. | N.a. |
| South America | 1 | 66 | 0.0000 | 0.0000 – 1.0000 | N.a. | N.a. |
| **Sample type** | Test for between groups difference: Q = 5.75; p = 0.056 | | | | | |
| Chronic | 9 | 2063326 | 0.0355 | 0.0108 – 0.1102 | 99.8 | 0.00 |
| FEP | 5 | 748 | 0.0359 | 0.0035 – 0.2806 | 85.2 | 0.02 |
| Not specified / Mixed sample | 13 | 357628 | 0.0081 | 0.0031 – 0.0209 | 98.8 | 0.00 |
| **Setting** | Test for between groups difference: Q = 8.68; p = 0.013* | | | | | |
| Inpatient | 8 | 2030 | 0.0594 | 0.0178 – 0.1804 | 93.7 | 0.00 |
| Outpatient | 5 | 19250 | 0.0074 | 0.0015 – 0.0347 | 94.0 | 0.00 |
| Not specified / Mixed sample | 15 | 2400422 | 0.0130 | 0.0054 – 0.0307 | 99.7 | 0.00 |
| **HBV Prevalence** | | | | | | |
| **Setting** | Test for between groups difference: Q = 18.93; p < 0.001* | | | | | |
| Inpatient | 4 | 836 | 0.0981 | 0.0699 – 0.1360 | N.a. | N.a. |
| Outpatient | 3 | 251 | 0.0040 | 0.0001 – 0.2297 | N.a. | N.a. |
| Not specified / Mixed sample | 7 | 40235 | 0.0292 | 0.0107 – 0.0771 | 99.1 | 0.00 |
| **HCV Prevalence** | | | | | | |
| **Setting** | Test for between groups difference: Q = 1.60; p = 0.450 | | | | | |
| Inpatient | 3 | 1185 | 0.0473 | 0.0091 – 0.2121 | 88.3 | 0.04 |
| Outpatient | 4 | 349 | 0.0159 | 0.0003 – 0.4793 | N.a. | N.a. |
| Not specified / Mixed sample | 13 | 144792 | 0.0265 | 0.0116 – 0.0592 | 99.4 | 0.00 |

CI Confidence Interval; HIV Human Immunodeficiency Virus; HBV Hepatitis B Virus; HCV Hepatitis C Virus; FEP First Episode Psychosis

**Table S10:** Subgroup analyses for the studied sex outcomes.

|  | **No. Studies** | **Sample size** | **Proportion** | **95% CI** | **Heterogeneity** | |
| --- | --- | --- | --- | --- | --- | --- |
|  |  |  |  |  | **I^2^ (%)** | ***p*** |
| **Proportion of schizophrenia patients in a stable romantic relationship** | | | | | | |
| **Continent** | Test for between groups difference: Q = 7.90; p = 0.095 | | | | | |
| North America | 4 | 436 | 0.2488 | 0.0218 – 0.8314 | 97.1 | 0.00 |
| Africa | 2 | 537 | 0.4219 | 0.0885 – 0.8457 | 72.8 | 0.03 |
| Europe | 7 | 390 | 0.2328 | 0.1163 – 0.4115 | 87.8 | 0.00 |
| Oceania | 2 | 136 | 0.3175 | 0.0048 – 0.9784 | N.a. | N.a. |
| Asia | 5 | 628 | 0.9037 | 0.0145 – 0.9998 | 93.9 | 0.00 |
| **Setting** | Test for between groups difference: Q = 4.01; p = 0.134 | | | | | |
| Inpatient | 5 | 579 | 0.1743 | 0.0587 – 0.4166 | 94.2 | 0.00 |
| Outpatient | 12 | 1326 | 0.5441 | 0.1836 – 0.8636 | 92.8 | 0.00 |
| Not specified / Mixed sample | 3 | 222 | 0.2820 | 0.0569 – 0.7187 | 88.7 | 0.00 |
| **Proportion of sexually active schizophrenia patients** | | | | | | |
| **Continent** | Test for between groups difference: Q = 2.86; p = 0.413 | | | | | |
| North America | 7 | 1498 | 0.3302 | 0.2268 – 0.4531 | 92.2 | 0.00 |
| Africa | 1 | 108 | 0.4167 | 0.3276 – 0.5483 | N.a. | N.a. |
| Europe | 6 | 309 | 0.4258 | 0.3117 – 0.5483 | 62.9 | 0.06 |
| Asia | 2 | 377 | 0.4191 | 0.1607 – 0.7310 | N.a. | N.a. |
| **Setting** | Test for between groups difference: Q = 4.01; p = 0.134 | | | | | |
| Inpatient | 3 | 388 | 0.4149 | 0.3128 – 0.5249 | N.a. | N.a. |
| Outpatient | 9 | 1414 | 0.3525 | 0.2534 – 0.4662 | 90.5 | 0.00 |
| Mix | 4 | 490 | 0.4408 | 0.3711 – 0.5129 | N.a. | N.a. |

CI Confidence Interval

**Figure S3:** Funnel plots for publication bias.


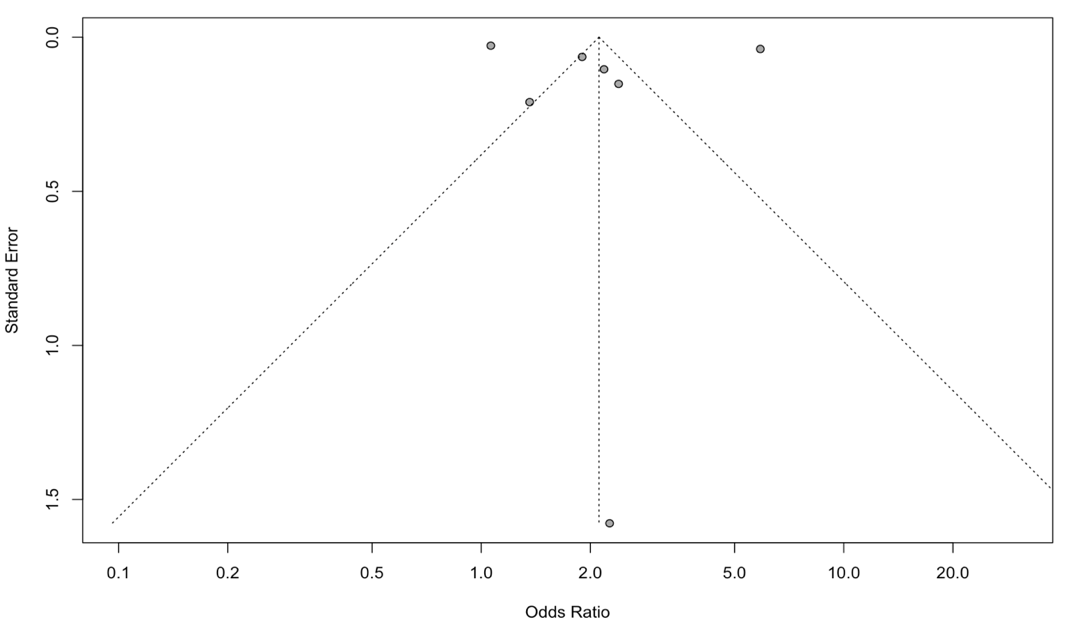

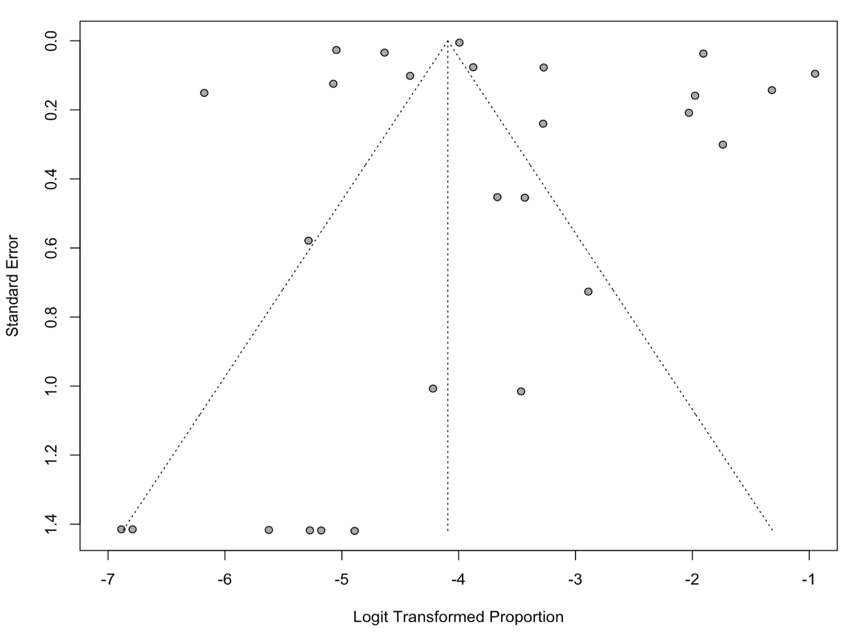


**Figure S3-B**. HIV Odds Ratio

**Figure S3-A**. HIV Prevalence


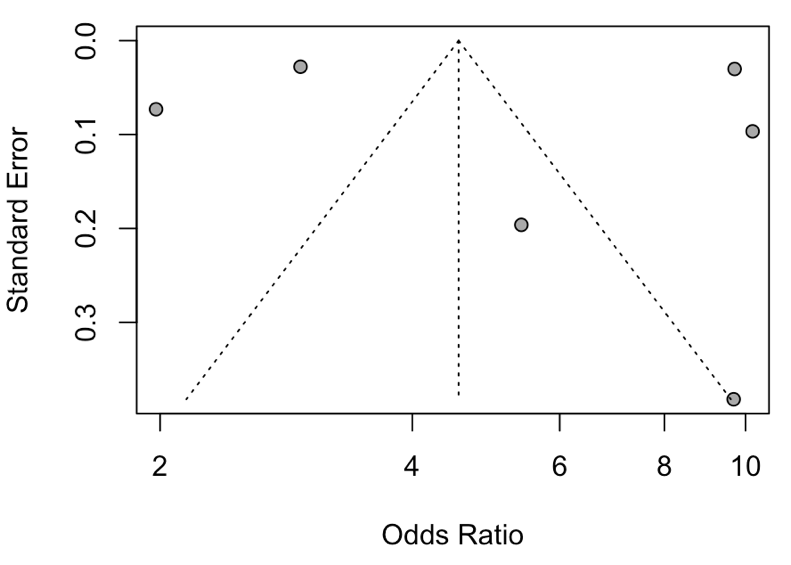

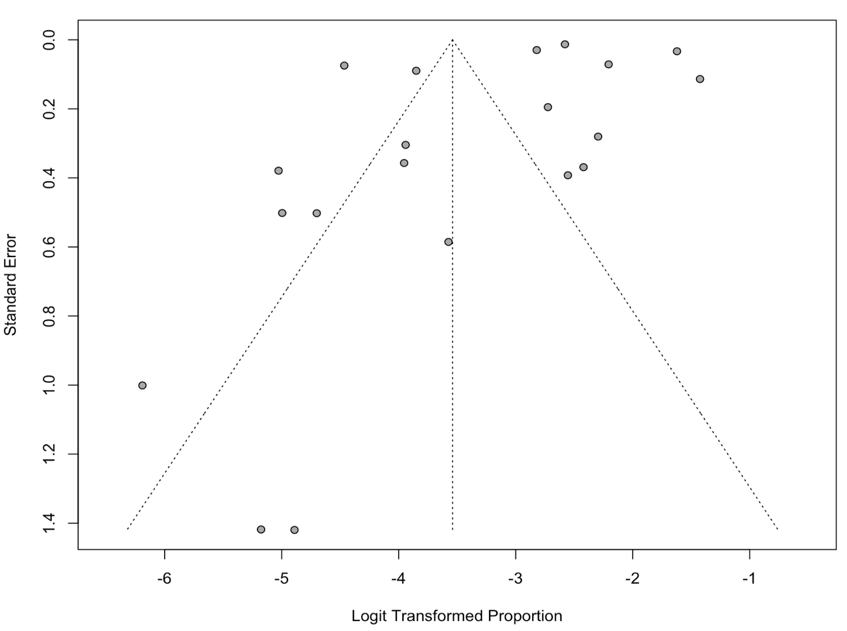


**Figure S3-D**. HCV Odds Ratio

**Figure S3-C**. HCV Prevalence


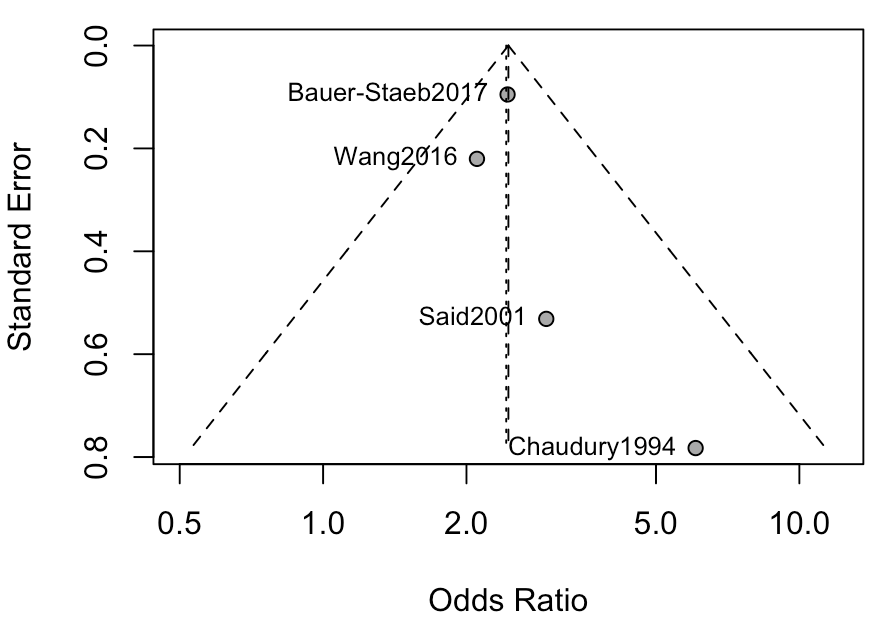

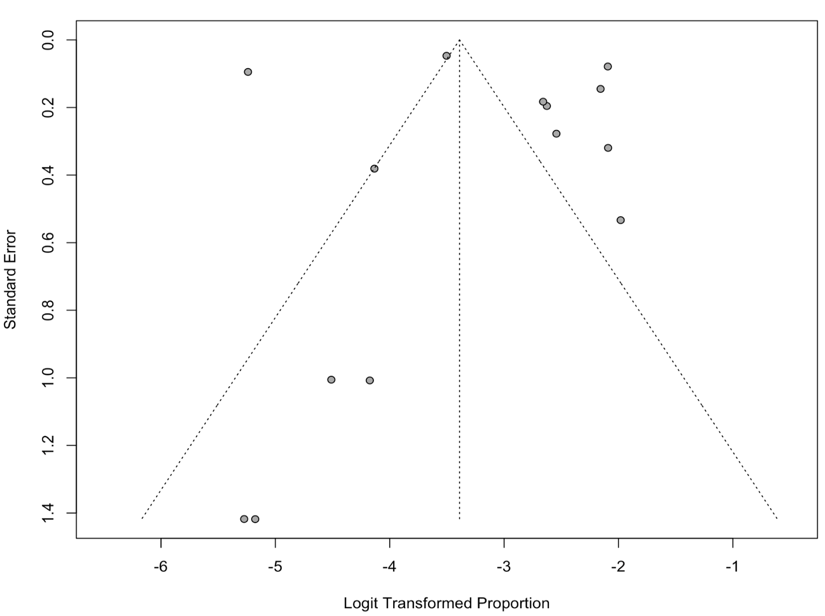


**Figure S3-F**. HBV Odds Ratio

**Figure S3-E**. HBV Prevalence


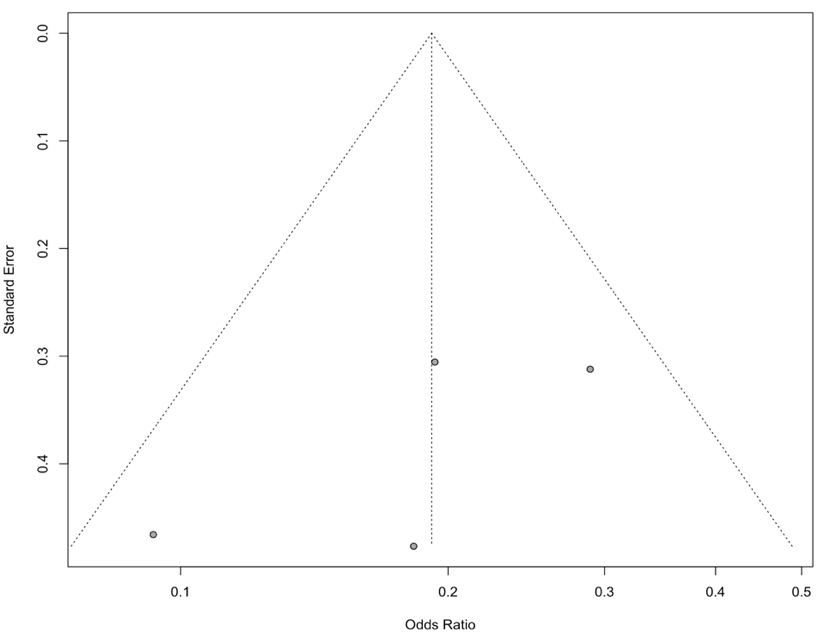

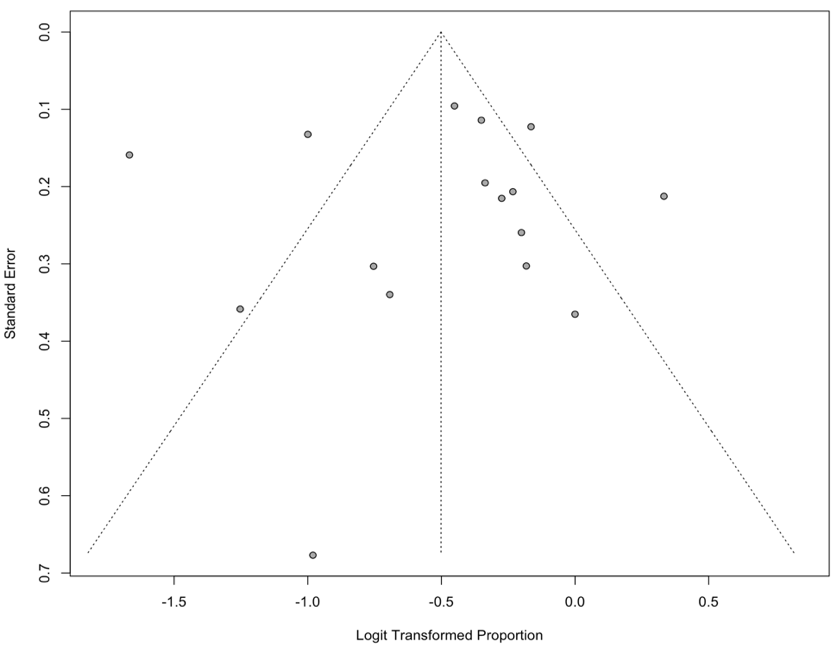

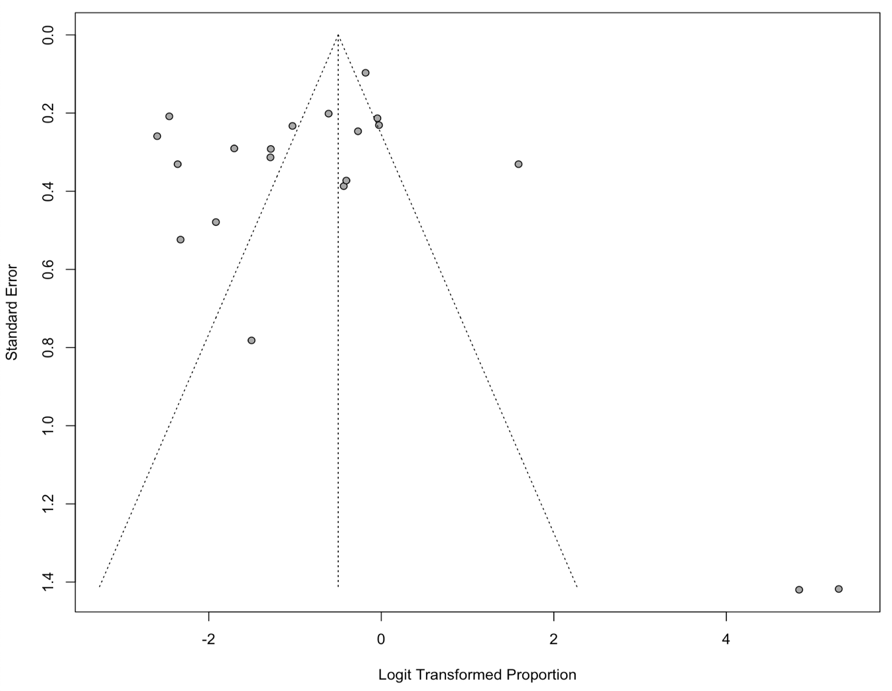

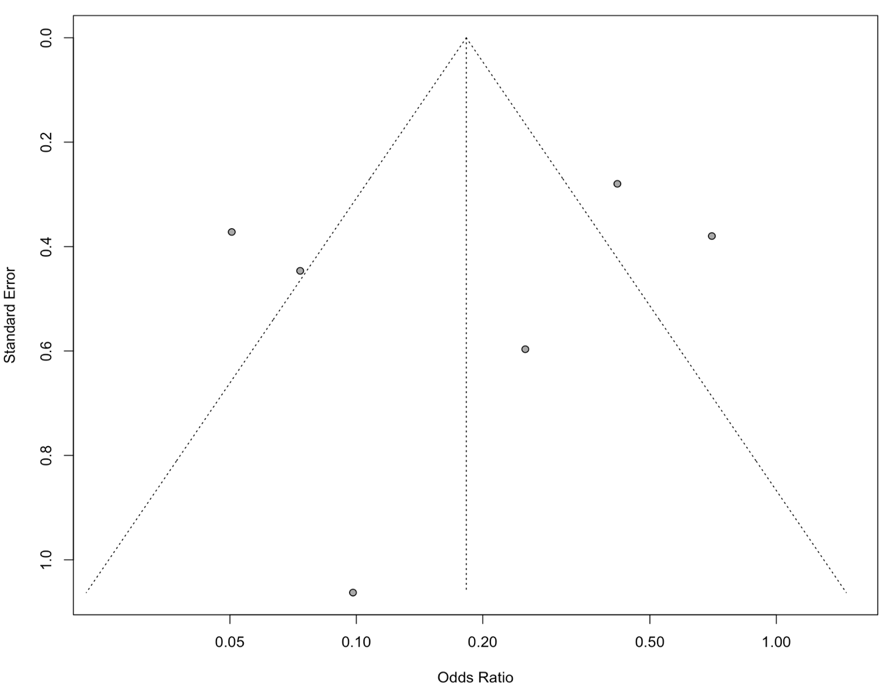


**Figure S3-J**. Odds ratio for being sexually active.

**Figure S3-I**. Proportion of sexually active patients.

**Figure S3-H**. Odds ratio for being a stable romantic relationship.

**Figure S3-G**. Proportion of patients in a stable romantic relationship.

REFERENCES

1. KLAF FS. Female homosexuality and paranoid schizophrenia*. Archives of general psychiatry*. 1961;4(1):84-86. doi: 10.1001/archpsyc.1961.01710070086011.

2. Brown A, Lubman DI, Paxton SJ. Reducing sexually-transmitted infection risk in young people with first-episode psychosis*. International journal of mental health nursing*. 2011;20(1):12-20. doi: 10.1111/j.1447-0349.2010.00700.x.

3. de Jager J, McCann E. Psychosis as a Barrier to the Expression of Sexuality and Intimacy: An Environmental Risk?*. Schizophrenia bulletin*. 2017;43(2):236-239. doi: 10.1093/schbul/sbw172.

4. A[degrees]ncedere A, Kucuk L. Sexual Life and Associated Factors in Psychiatric Patients*. Sex Disabil*. 2017;35(1):89-106. doi: 10.1007/s11195-017-9475-y.

5. Simiyon M, Chandra PS, Desai G. Sexual dysfunction among women with Schizophrenia—A cross sectional study from India*. Asian journal of psychiatry*. 2016;24:93-98. doi: 10.1016/j.ajp.2016.08.022.

6. Ma M, Chao J, Hung J, Sung S, Chao IC. Sexual Activity, Sexual Dysfunction, and Sexual Life Quality Among Psychiatric Hospital Inpatients With Schizophrenia*. Journal of sexual medicine*. 2018;15(3):324-333. doi: 10.1016/j.jsxm.2018.01.008.

7. Hannachi N, El Kissi Y, Samoud S, et al. High prevalence of Human Herpesvirus 8 in schizophrenic patients*. Psychiatry research*. 2014;216(2):192-197. doi: 10.1016/j.psychres.2013.12.035.

8. Carey MP, Carey KB, Maisto SA, Gleason JR, Gordon CM, Brewer KK. HIV-risk behavior among outpatients at a state psychiatric hospital: Prevalence and risk modeling*. Behavior therapy*. 1999;30(3):389-406. doi: 10.1016/S0005-7894(99)80017-3.

9. Raja M, Azzoni A. Sexual behavior and sexual problems among patients with severe chronic psychoses*. European psychiatry*. 2003;18(2):70-76. doi: 10.1016/S0924-9338(03)00009-9.

10. GRASSI L, PAVANATI M, CARDELLI R, FERRI S, PERON L. HIV-risk behaviour and knowledge about HIV/AIDS among patients with schizophrenia*. Psychological medicine*. 1999;29(1):171-179. doi: 10.1017/S0033291798007818.

11. MCLENNAN JD, GANGULI R. Family planning and parenthood needs of women with severe mental illness: clinicians' perspective*. Community mental health journal*. 1999;35(4):369-380. doi: 10.1023/A:1018770109042.

12. Bai Y, Huang Y, Lin C, Chen J. Emerging homosexual conduct during hospitalization among chronic schizophrenia patients*. Acta psychiatrica Scandinavica*. 2000;102(5):350-353. doi: 10.1034/j.1600-0447.2000.102005350.x.

13. Carey MP, Carey KB, Maisto SA, Gordon CM, Vanable PA. Prevalence and Correlates of Sexual Activity and HIV-Related Risk Behavior Among Psychiatric Outpatients*. Journal of consulting and clinical psychology*. 2001;69(5):846-850. doi: 10.1037/0022-006X.69.5.846.

14. McCann E. The expression of sexuality in people with psychosis: breaking the taboos*. Journal of advanced nursing*. 2000;32(1):132-138. doi: 10.1046/j.1365-2648.2000.01452.x.

15. Carey M, Carey K, Maisto S, Schroder K, Vanable P, Gordon C. HIV Risk Behavior Among Psychiatric Outpatients: Association With Psychiatric Disorder, Substance Use Disorder, and Gender*. The journal of nervous and mental disease*. 2004;192(4):289-296. doi: 10.1097/01.nmd.0000120888.45094.38.

16. Brown E, Castagnini E, Langstone A, et al. High‐risk sexual behaviours in young people experiencing a first episode of psychosis*. Early intervention in psychiatry*. 2023;17(2):159-166. doi: 10.1111/eip.13301.

17. MCCANN E. The sexual and relationship needs of people who experience psychosis: quantitative findings of a UK study*. Journal of psychiatric and mental health nursing*. 2010;17(4):295-303. doi: 10.1111/j.1365-2850.2009.01522.x.

18. Brown AP, Lubman DI, Paxton SJ. Psychosocial Risk Factors for Inconsistent Condom Use in Young People with First Episode Psychosis*. Community Ment Health J*. 2011;47(6):679-687. doi: 10.1007/s10597-011-9370-4.

19. Gonzalez-Torres MA, Salazar MA, Inchausti L, et al. Lifetime Sexual Behavior of Psychiatric Inpatients*. Journal of sexual medicine*. 2010;7(9):3045-3056. doi: 10.1111/j.1743-6109.2010.01795.x.

20. Acuña MJ, Martín JC, Graciani M, Cruces A, Gotor F. A Comparative Study of the Sexual Function of Institutionalized Patients with Schizophrenia*. Journal of sexual medicine*. 2010;7(10):3414-3423. doi: 10.1111/j.1743-6109.2010.01832.x.

21. Hariri AG, Karadag F, Gokalp P, Essizoglu A. Risky Sexual Behavior among Patients in Turkey with Bipolar Disorder, Schizophrenia, and Heroin Addiction*. Journal of sexual medicine*. 2011;8(8):2284-2291. doi: 10.1111/j.1743-6109.2011.02282.x.

22. Kazour F, Obeid S, Hallit S. Sexual desire and emotional reactivity in chronically hospitalized Lebanese patients with schizophrenia*. Perspectives in psychiatric care*. 2020;56(3):502-507. doi: 10.1111/ppc.12455.

23. COURNOS F, GUIDO JR, SIOBHAN COOMARASWAMY, MEYER-BAHLBURG H, SUGDEN R, HORWATH E. Sexual activity and risk of HIV infection among patients with schizophrenia*. The American journal of psychiatry*. 1994;151(2):228-232. doi: 10.1176/ajp.151.2.228.

24. Bianco CL, Pratt SI, Ferron JC. Deficits in Sexual Interest Among Adults With Schizophrenia: Another Look at an Old Problem*. Psychiatric services (Washington, D.C.)*. 2019;70(11):1000-1005. doi: 10.1176/appi.ps.201800403.

25. Lindamer LA, Buse DC, Auslander L, Unützer J, Bartels SJ, Jeste DV. A Comparison of Gynecological Variables and Service Use Among Older Women With and Without Schizophrenia*. Psychiatric services (Washington, D.C.)*. 2003;54(6):902-904. doi: 10.1176/appi.ps.54.6.902.

26. MILLER LJ, FINNERTY M. Sexuality, pregnancy, and childrearing among women with schizophrenia- spectrum disorders*. Psychiatric services (Washington, D.C.)*. 1996;47(5):502-506. doi: 10.1176/ps.47.5.502.

27. Negash B, Asmamewu B, Alemu WG. Risky sexual behaviors of schizophrenic patients: a single center study in Ethiopia, 2018*. BMC Research Notes*. 2019;12(1):635. doi: 10.1186/s13104-019-4673-6.

28. Wright ER, Wright DE, Perry BL, Foote-Ardah CE. Stigma and the Sexual Isolation of People with Serious Mental Illness*. Social problems (Berkeley, Calif.)*. 2007;54(1):78-98. doi: 10.1525/sp.2007.54.1.78.

29. Miller LJ, Finnerty M. Family planning knowledge, attitudes and practices in women with schizophrenic spectrum disorders*. Journal of psychosomatic obstetrics and gynaecology*. 1998;19(4):210-217. doi: 10.3109/01674829809025699.

30. Shaikh RAK, Ghogare AS, Prasad P, Deshmukh S. A Cross-sectional Study of Antipsychotic Drugs Induced Sexual Dysfunction Among Married Males with Remitted Schizophrenia Attending Tertiary Health Care Centre from Central India*. Journal of clinical and diagnostic research*. 2021;15(3):VC01-VC07. doi: 10.7860/JCDR/2021/46625.14608.
